# Construction of a clickable probe‐based protein chip platform for discovering covalent mIDH1 inhibitors from natural medicinal extracts

**DOI:** 10.1002/imt2.70107

**Published:** 2026-02-12

**Authors:** Zhao Cui, Jiameng Li, Caifeng Li, Mo Sun, Wei Liu, Xuxia Cao, Shiwen Deng, Junxian Cao, Hongjun Yang, Peng Chen

**Affiliations:** ^1^ Laboratory of Cardiovascular Diseases, Xiyuan Hospital of China Academy of Chinese Medical Sciences Beijing China; ^2^ Experimental Research Center, China Academy of Chinese Medical Sciences Beijing China; ^3^ National Clinical Research Center for TCM Cardiology, Xiyuan Hospital of China Academy of Chinese Medical Sciences Beijing China; ^4^ The First Affiliated Hospital of Guangzhou Medical University Guangzhou China; ^5^ CAS Key Laboratory for Biomedical Effects of Nanomaterials and Nanosafety, Chinese Academy of Sciences and National Center for Nanoscience and Technology of China Beijing China; ^6^ Jiangxi Province Key Laboratory of Traditional Chinese Medicine Pharmacology, Institute of Traditional Chinese Medicine Health Industry, China Academy of Chinese Medical Sciences Nanchang China; ^7^ School of Biological Sciences, Georgia Institute of Technology Atlanta USA; ^8^ BinZhou Medical University Yantai China

**Keywords:** bioorthogonal click chemistry, covalent active molecules, flavokawain c, medicinal plants, mutant isocitrate dehydrogenase 1 inhibitor, natural product screening, protein microarray

## Abstract

The early discovery of covalent drugs is frequently inspired by, or derived from, natural sources, with such compounds often showing favorable safety profiles and a comparatively lower risk of clinical failure. However, a straightforward, high‐throughput technique for screening covalent‐binding molecules directly from complex medicinal plant extracts remains unavailable. In this study, we introduce an integrated strategy that combines protein microarrays with bioorthogonal click chemistry (Ccc‐Chip). This platform includes a differential scanning fluorimetry (DSF)‐based pre‐screening step to enhance efficiency, with the Ccc‐Chip serving as the core confirmation tool. It provides simple and intuitive readouts, enabling synchronous, high‐throughput screening of covalent ligands targeting multiple proteins through detection of their competitive binding with cysteine‐reactive probes. To validate the approach, we constructed a mutant isocitrate dehydrogenase 1 (mIDH1) protein microarray and used the integrated workflow to screen 110 medicinal plants. Our results led to the identification of flavokawain C (Flc), a covalent inhibitor of mIDH1, from *Piper methysticum* Forst. Subsequent in vivo experiments showed that Flc significantly reduced 2‐hydroxyglutarate (2‐HG) levels in an mIDH1‐driven orthotopic tumor model and enhanced CD8⁺ T cell activity. Notably, when combined with a programmed cell death protein 1 (PD‐1) blocking antibody, Flc synergistically augmented antitumor immunity, resulting in suppressed tumor growth. This work not only supports the high‐throughput utility of the Ccc‐Chip strategy but also provides a practical framework for combining bioorthogonal labeling with protein microarray technology, facilitating the discovery of bioactive covalent molecules from plant sources for challenging therapeutic targets.

## Highlights

- Integrate and develop a bioorthogonal click chemistry‐protein chip platform for the label‐free and high‐throughput screening​ of covalent inhibitors directly from complex plant extracts.
- Flc as a covalent inhibitor of mIDH1 from 110 medicinal plant extracts; it significantly reduces the level of the oncogenic metabolite 2‐HG, and its combination with PD‐1 antibodies synergistically enhances anti‐tumor immunity.
- The platform is scalable, with flexibility for target proteins and specific probes, providing a solution with high sensitivity and specificity for mining covalent active molecules against refractory targets from natural medicinal plant sources.

## Graphical abstract

In this study, we develop a flexible Ccc‐Chip (protein microarray + bioorthogonal click chemistry) for high‐throughput screening of natural covalent active molecules from medicinal plant extracts, identifying flavokawain C from *Piper methysticum Forst* as a novel covalent mIDH1 inhibitor.

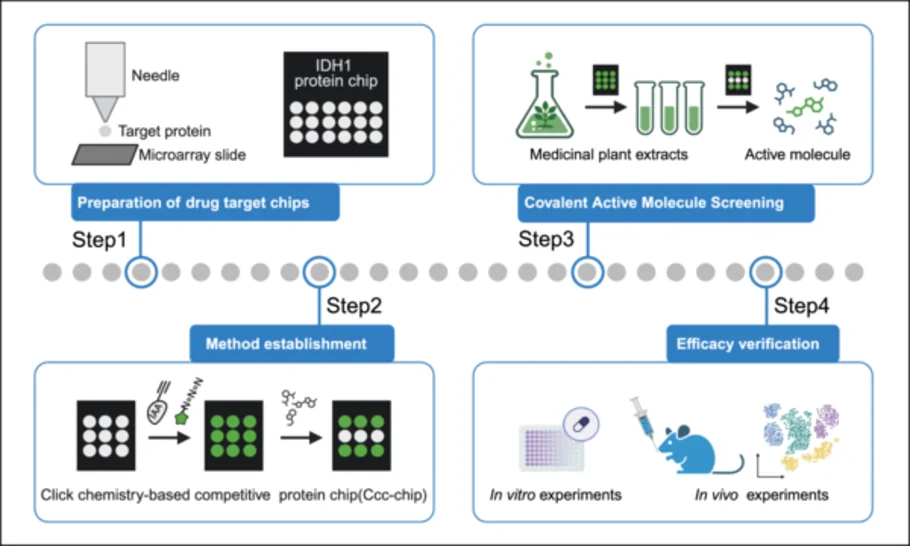

## INTRODUCTION

Covalent drugs play a pivotal role in treating a variety of diseases, including cancer and infectious diseases [1, 2]. Natural products represent a major source for covalent drug discovery, with many marketed covalent drugs, such as penicillin, aspirin, and orlistat, being directly or indirectly derived from natural lead compounds [3, 4, 5, 6]. Compared to fully synthetic covalent drugs, naturally derived covalent molecules often demonstrate superior safety profiles and favorable drug‐like properties, accompanied by a comparatively lower risk of clinical development failure. Liquid chromatography‐electrospray ionization mass spectrometry (LC‐ESI‐MS) is a widely used technique in covalent drug discovery. However, conventional LC‐ESI‐MS methods generally require chromatographic separation for each individual sample, which limits analytical throughput [7]. Moreover, the common practice of compound pooling complicates the analytical workflow further, posing significant challenges when applied to the complex mixtures found in medicinal plant extracts [8, 9]. Consequently, the development of novel technologies and strategies capable of rapid and accurate screening of covalent drugs from medicinal plants remains a critical need to propel this field forward.

Bioorthogonal labeling technology enables the visualization of ligand–protein interactions by conjugating fluorescent reporter molecules to target proteins through copper(I)‐catalyzed azide‐alkyne cycloaddition click reactions [10]. This strategy has been successfully applied to a variety of drug molecules and natural active compounds, including metformin, artemisinin, dasatinib, and leucosceptrine F, achieving specific labeling and identification of their corresponding target proteins [10, 11, 12, 13]. However, this method typically requires prior chemical modification of the ligand molecules, which substantially limits its applicability in high‐throughput screening of complex extract systems or large compound libraries. While competitive binding‐based strategies, where known covalent probes compete with test molecules from extracts for target protein binding sites, offer theoretical potential for identifying specific covalent protein ligands in complex mixtures, traditional gel‐based or cellular fluorescence imaging methods suffer from low throughput [14]. Therefore, there is an urgent need for innovative approaches that enable high‐throughput screening of large numbers of candidate drugs within a single experiment. Protein microarray technology leverages the principle of specific biomolecular interactions, wherein large numbers of protein molecules are immobilized in a high‐density microarray format on solid‐phase supports, creating a high‐throughput platform for functional protein analysis [15, 16, 17, 18]. This technology enables parallel detection of the binding behaviors of thousands of proteins, requiring only minimal sample volumes while facilitating rapid and highly sensitive screening. Its inherent high‐throughput capacity makes it particularly well‐suited for screening active compounds from natural extracts using competitive binding strategies. To address existing methodological limitations, we developed an integrated screening system combining bioorthogonal labeling with protein microarray technology (termed Ccc‐Chip) to efficiently identify active components in complex natural extracts. To validate this system, we selected mutant isocitrate dehydrogenase 1 (mIDH1) as a model target. This mutant represents a common metabolic enzyme alteration in tumors, catalyzing the production of the oncometabolite D‐2‐hydroxyglutarate (2‐HG). Given the mutational diversity of mIDH1 and associated clinical drug resistance challenges, we constructed a protein microarray encompassing multiple prevalent IDH1 mutants. This platform enables efficient evaluation of the inhibitory potential of natural extract components against various mutant forms, thereby facilitating the discovery of covalent mIDH1 inhibitors.

In this study, we established a protein microarray‐based screening platform for covalent molecules, utilizing the cysteine‐targeting covalent probe iodoacetamide‐alkyne (IAA‐yne) to quantify the occupancy of natural ligands on mIDH1. The Ccc‐Chip platform served as the core screening tool within this integrated system. Screening 110 natural extracts led to the identification of flavokawain C (Flc), a covalent inhibitor of mIDH1 derived from *Piper methysticum* Forst. Flc was validated to inhibit mIDH1 enzymatic activity, reduce in situ 2‐HG production, and enhance CD8^+^ T cell activity. Furthermore, in combination with a PD‐1 antibody, Flc demonstrated enhanced suppression of mIDH1‐driven tumor growth. This study successfully integrates the scalability of protein microarrays with the specificity of bioorthogonal chemistry into the Ccc‐Chip platform, enabling bidirectional high‐throughput screening. As detailed in Table S1, this strategy overcomes key limitations of existing methods by allowing the direct screening of complex natural extracts for covalent inhibitors without pre‐modification, thereby establishing an efficient and accessible route to lead discovery from natural sources.

## RESULTS

### The establishment of the Ccc‐Chip methodology on mIDH1 protein chip

As illustrated in Figure 1A, our strategy determines whether a test molecule binds covalently by assessing its competition with the cysteine‐targeting probe IAA‐yne for binding to target proteins immobilized on a protein array. In the absence of competition, the fluorescence signal corresponding to the target protein, reflecting IAA‐yne binding, indicates that the test molecule does not bind covalently. Conversely, a significant reduction in this signal signals that the test molecule competes with IAA‐yne, thus acting as a covalent binder. To validate this approach, we first demonstrated on P50, AKT1, and KRAS^G12C^ protein arrays that IAA and IAA‐yne compete for binding to the target proteins (Figure 1B–D). Next, we selected known covalent binders for each target, andrographolide for P50, borussertib for AKT1, and AMG510 for KRAS^G12C^, and confirmed through in‐gel fluorescence click chemistry that these molecules effectively compete with IAA‐yne for their respective binding sites (Figure S1). Finally, protein array validation experiments showed that andrographolide, borussertib, and AMG510 each competed with IAA‐yne for binding to their corresponding target proteins. Additionally, a mixture of andrographolide and borussertib simultaneously competed with IAA‐yne for binding to both P50 and AKT1 (Figure 1C,D).

**Figure 1:**
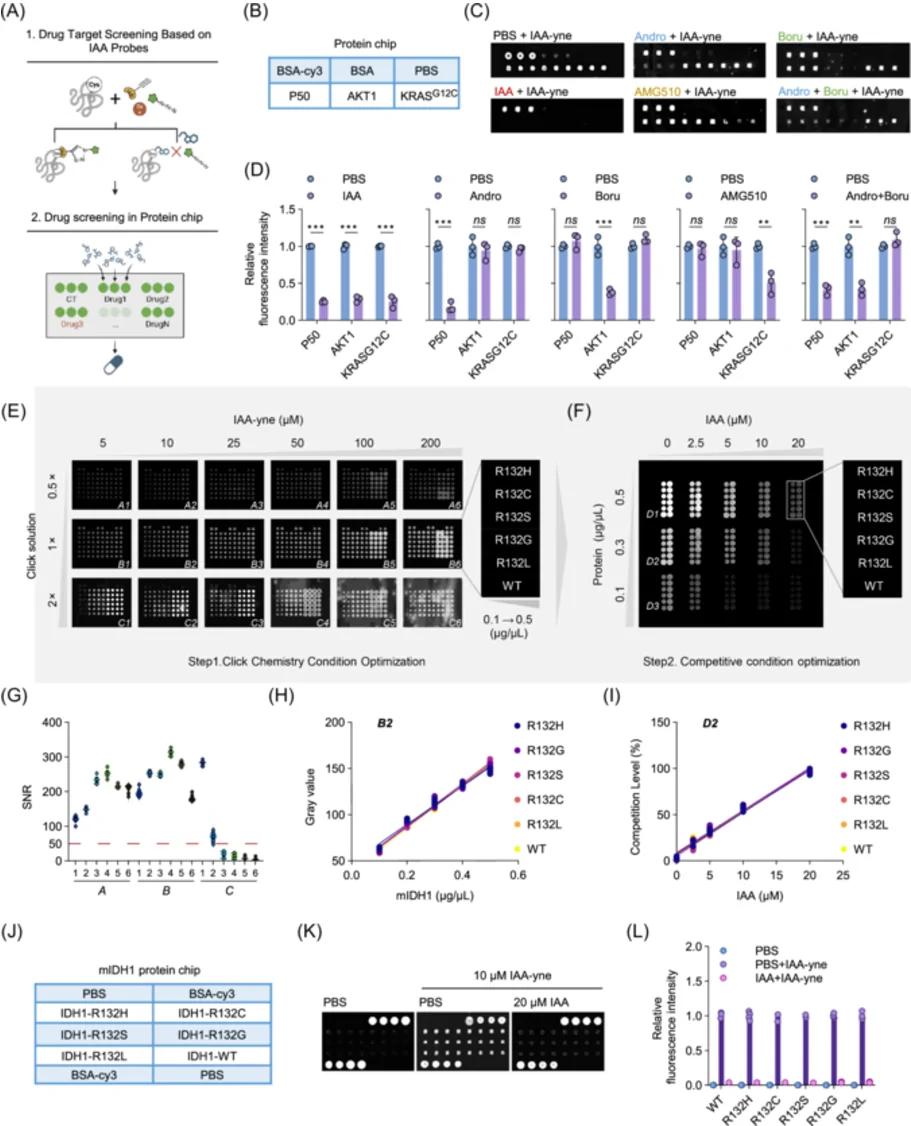
Construction and optimization of an mIDH1 protein microarray for Ccc‐Chip screening. (A) Schematic illustration of the Ccc‐Chip experimental principle based on cysteine‐targeted IAA. (B) Design of a protein microarray containing recombinant P50, AKT1, and KRAS^G12C^ proteins. (C) Ccc‐Chip analysis of the competitive binding of IAA and IAA‐yne to recombinant P50, AKT1, and KRAS^G12C^ proteins (*n* = 3). (D) Ccc‐Chip analysis of the competitive binding between cysteine‐targeted covalent molecules and IAA‐yne to recombinant P50, AKT1, and KRAS^G12C^ proteins (*n* = 3). (E) Chip matrix used for combined optimization of IAA‐yne concentration and click‐chemistry reagent concentration. mIDH1 protein concentrations were 0.1, 0.2, 0.3, 0.4, and 0.5 μg/μL (*n* = 2). (F) Screening matrix for optimization of mIDH1 protein concentration (*n* = 2). (G) SNR of mIDH1 signals under varying IAA‐yne and click‐chemistry reagent concentrations (*n* = 6). (H) Linear regression and correlation between protein signal intensity and mIDH1 protein concentration under 10 μM IAA‐yne and 1× click‐chemistry conditions (*n* = 4). R132H‐*R* ^2^ = 0.978; R132G‐*R* ^2^ = 0.977; R132S‐*R* ^2^ = 0.983; R132C‐*R* ^2^ = 0.983; R132L‐*R* ^2^ = 0.979; WT‐*R* ^2^ = 0.991. (I) Linear regression and correlation analysis of competitive binding between IAA and IAA‐yne at an mIDH1 protein concentration of 0.3 μg/μL (*n* = 4). R132H‐*R* ^2^ = 0.987; R132G‐*R* ^2^ = 0.982; R132S‐*R* ^2^ = 0.986; R132C‐*R* ^2^ = 0.987; R132L‐*R* ^2^ = 0.982; WT‐*R* ^2^ = 0.986. (J) Design of a protein microarray containing recombinant wild‐type and mutant IDH1 proteins. (K, L) Validation of cysteine‐specific competitive binding on the IDH1 protein chip using IAA (*n* = 4). Data are represented as mean ± SEM. *p < 0.05; **p < 0.01; ***p < 0.001; *ns*, not significant. IAA, iodoacetamide; IAA‐yne, iodoacetamide‐alkyne; BSC‐cy3, bovine serum albumin‐cyanine 3; P50, NF‐κB p50 subunit; AKT1, AKT serine/threonine kinase 1; KRAS^G12C^, KRAS‐G12C mutant; Andro, andrographolide; Boru, borussertib; WT, wild type; mIDH1, mutant IDH1; SNR, signal‐to‐noise ratio. See also Figures S1 and S2.

Our research group has long focused on developing natural bioactive molecules targeting both wild‐type and mutant IDH1 [19, 20]. Despite the approval of several clinical mIDH1 inhibitors, challenges such as incomplete coverage of mIDH1 subtypes, acquired drug resistance, and off‐target toxicity remain unresolved. Given the high prevalence of IDH1 mutations in tumors and the significant unmet clinical needs, we constructed an mIDH1 protein matrix to optimize conditions for covalent molecule screening (Figure 1E,F). First, as shown in Figure 1E, we screened for the optimal combination of IAA‐yne concentration and click chemistry solution concentration to maximize click chemistry labeling efficiency on the mIDH1 protein microarray. We initially evaluated the signal‐to‐noise ratio (SNR) under various reaction conditions (Figure S2A), finding that all except conditions C4, C5, and C6 demonstrated favorable SNR values greater than 50 (Figure 1G). Further analysis of the linear correlation between mIDH1 protein concentration and signal intensity revealed strong linearity under conditions B1, B2, A1, and A2 (Figure S2B,C, Figure 1H). Notably, condition B2 (10 μM IAA‐yne with 1× click chemistry solution) showed the strongest linear correlation and was therefore selected as the optimal click chemistry reaction condition for the mIDH1 protein microarray (Figure 1H). Using this optimal click chemistry condition, we next screened for the ideal mIDH1 protein concentration for competitive binding assays with IAA (Figure 1F). Results demonstrated a strong linear correlation between the IAA/IAA‐yne competitive ratio and IAA concentration at an mIDH1 protein concentration of 0.3 μg/μL (Figure S2D, Figure 1I). Collectively, we established the optimal screening system for mIDH1‐targeting covalent competitive molecules: 10 μM IAA‐yne, 1× click chemistry solution, and 0.3 μg/μL mIDH1 protein. Using this system, we successfully constructed an mIDH1 protein microarray for covalent molecule screening, where 20 μM IAA nearly completely competed with IAA‐yne for binding to mIDH1 (Figure 1J–L). In summary, we developed a covalent molecule screening platform based on mIDH1 protein microarrays combined with click chemistry, providing a robust tool for subsequent covalent inhibitor screening.

### Identification of constituents targeting the mIDH1 protein from plant extracts via the Ccc‐Chip method

The screening of natural extracts targeting mIDH1 was conducted as outlined in the experimental workflow (Figure 2A). Initially, 110 medicinal plants were selected and subjected to preliminary extraction using 70% ethanol, followed by filtration to obtain crude extracts. These extracts were screened using differential scanning fluorimetry (DSF) and the Ccc‐Chip platform to identify candidates with potential targeting activity. Extracts that inhibited 2‐HG production in mIDH1 tumor cell lines were selected for further analysis. In DSF experiments, the standard melting temperature (*T*_m_) for each protein was first determined (Figure 2B). Using five covalent or non‐covalent molecules targeting mIDH1 as positive controls and the combination of scutellarin (Scu) and IDH1‐C297S as a negative control, we demonstrated that a 1.5°C shift in melting temperature (Δ*T*_m_) effectively distinguished true positive molecules from false positives (Figure S3). Thus, extracts inducing a *T*_m_ shift greater than 1.5°C for both mIDH1 variants were selected for subsequent screening rounds (Figure 2C, Table S2). These candidates underwent further DSF evaluation at multiple concentrations against both IDH1 wild‐type and mIDH1 proteins, and those showing concentration‐dependent *T*_m_ changes were advanced to Ccc‐Chip screening (Figure 2D). Ccc‐Chip screening identified extracts T30, T36, T56, T96, and T110 as competing with IAA‐yne for binding to mIDH1 (Figure 2E,F). These five extracts were subjected to functional validation, including cytotoxicity assays in TS603 and HT1080 cells, followed by assessment of their ability to inhibit 2‐HG production at a non‐cytotoxic concentration (10 μg/mL) (Figure 2G). The data demonstrated that only extract T96, derived from *P. methysticum* Forst., significantly inhibited 2‐HG production in a dose‐dependent manner from 5 to 40 μg/mL (Figure 2H,I). In contrast, extracts T30, T36, T56, and T110 showed no significant inhibition at the tested concentration (Figure 2H). Therefore, extract T96 was selected as a potential mIDH1‐targeting candidate and was subjected to further screening for covalent constituents.

**Figure 2:**
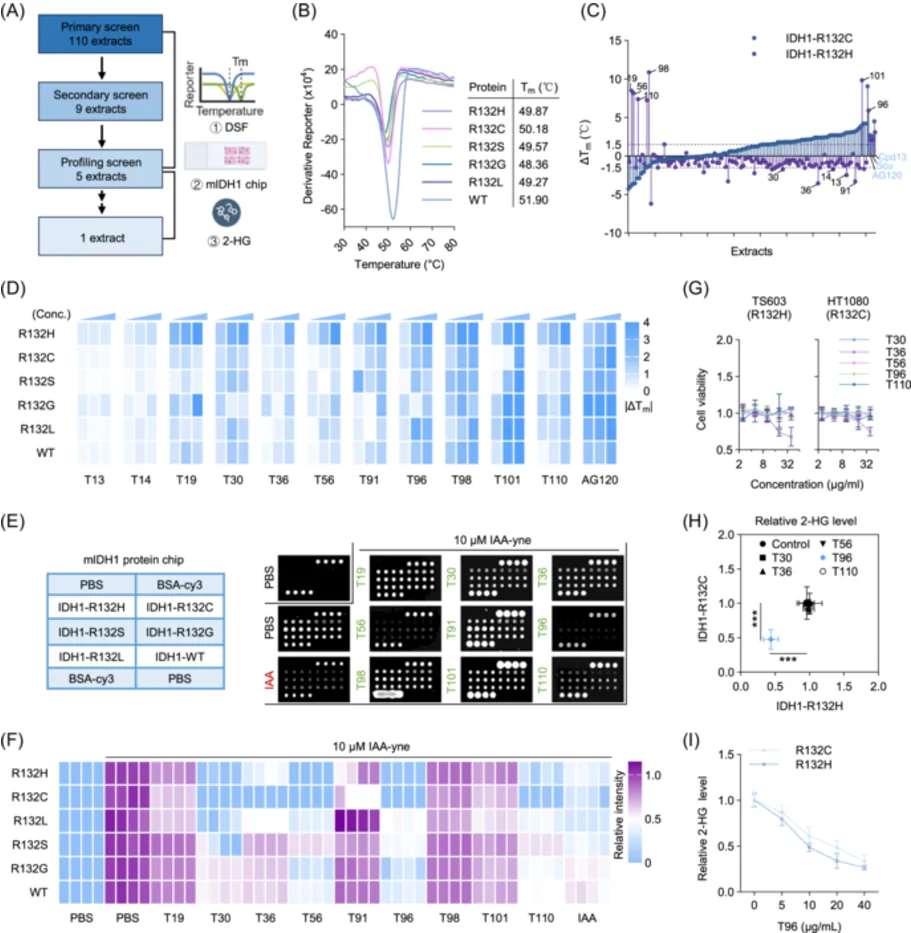
Ccc‐Chip‐based screening of medicinal plant extracts targeting mIDH1. (A) Workflow for screening medicinal plant extracts targeting IDH1. (B) Differential scanning fluorimetry (DSF) standard curves and Tm of recombinant wild‐type and mutant IDH1 proteins (*n* = 3). (C) Effects of medicinal plant extracts (20 μg/mL) on the *T*_m_ values of IDH1‐R132C and IDH1‐R132H. Candidates were selected based on an average absolute Δ*T*_m_ > 1.5°C across three replicates (*n* = 3). (D) Multi‐dose DSF screening of candidate medicinal plant extracts. AG120 was used as a positive control (*n* = 3). (E, F) Ccc‐Chip screening of medicinal plant extracts (20 μg/mL) targeting IDH1 (*n* = 4). (G) 24‐h proliferation curves of TS603 and HT1080 cells treated with candidate medicinal plant extracts (*n* = 3). (H) Effects of candidate liquid‐phase preparation components (10 μg/mL) on 2‐HG production in TS603 and HT1080 cells expressing IDH1‐R132H or IDH1‐R132C (*n* = 3). (I) Effects of T96 on 2‐HG production in TS603 and HT1080 cells expressing IDH1‐R132H or IDH1‐R132C (*n* = 3). Data are represented as mean ± SEM. *p < 0.05; **p < 0.01; ***p < 0.001; *ns*, not significant. IAA‐yne, iodoacetamide‐alkyne; Conc, concentration; T, extract; AG120, ivosidenib; DSF, differential scanning fluorimetry; mIDH1, mutant IDH1; *T*_m_, melting temperature; WT, wild type; 2‐HG, 2‐hydroxyglutarate. See also Table S2 and Figure S3.

As shown in Figure 3A, following identification of the candidate extract, preparative liquid chromatography was employed to fractionate the extract, and the resulting fractions were subsequently re‐screened using DSF and Ccc‐Chip assays. Specifically, the *P. methysticum* Forst. extract was separated into 35 consecutive fractions via preparative high‐performance liquid chromatography (Figure 3B). These fractions were then evaluated by DSF and Ccc‐Chip assays to identify those that altered the *T*_m_ of both wild‐type and mutant IDH1 proteins and competed with IAA‐yne for binding to the IDH1 protein (Figure 3C–E). Candidate fractions 7–11 and 18–22 were further validated in cellular assays. Among these, fractions 19 and 20 significantly inhibited 2‐HG production in TS603 and HT1080 cells at non‐cytotoxic concentrations (Figure 3F, Figure S4). Given that fractions 19 and 20 were consecutive in the chromatographic separation, they were combined for subsequent mass spectrometric analysis to identify their constituent compounds, facilitating the next stage of screening for covalent active molecules (Figure 3G).

**Figure 3:**
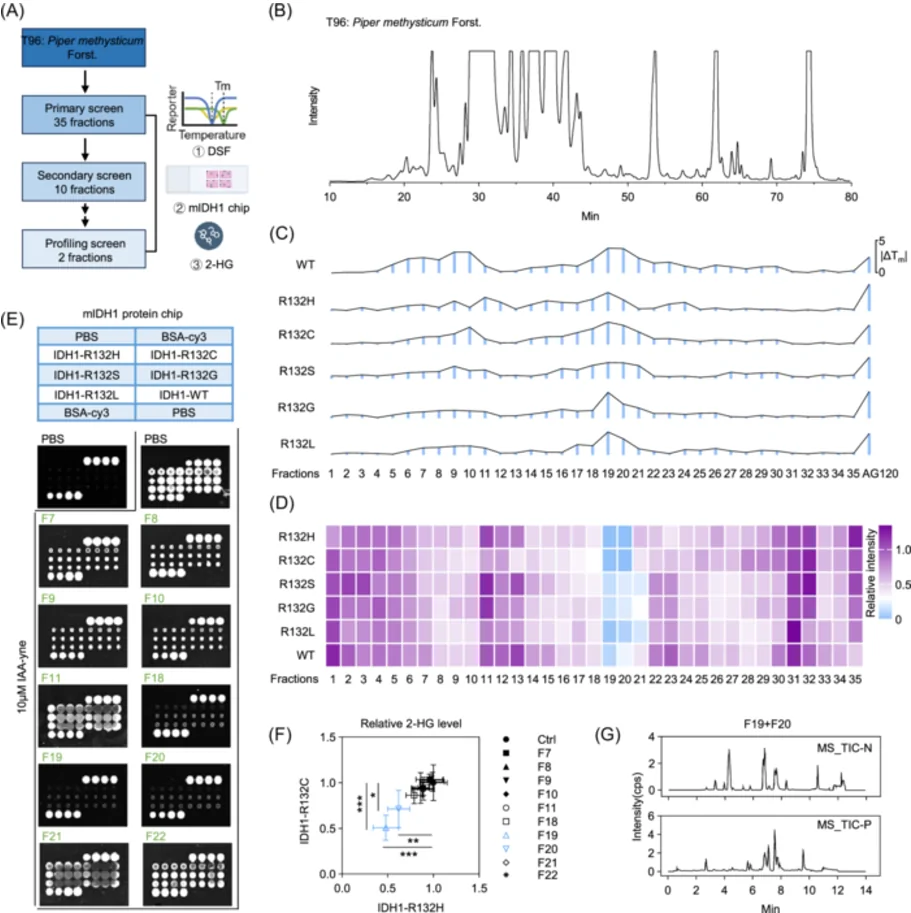
Identification of active IDH1‐targeting components from *P. methysticum* Forst extracts using Ccc‐Chip. (A) Workflow for screening active fractions of *P. methysticum* Forst targeting IDH1. (B) Preparative C18 HPLC chromatogram of the *P. methysticum* Forst extract (T96). (C) DSF evaluation of 35 preparative fractions (10 μg/mL) on the T_m of recombinant wild‐type and mutant IDH1 proteins. AG120 served as a positive control (*n* = 3). (D) Ccc‐Chip detection of liquid‐phase preparation components (10 μg/mL) targeting IDH1 in *P. methysticum* extracts (*n* = 4). (E) Signal profiles of protein chips for candidate liquid‐phase preparation components in Ccc‐Chip assays (*n* = 4). (F) Effects of candidate liquid‐phase preparation components (10 μg/mL) on 2‐HG production in TS603 and HT1080 cells expressing IDH1‐R132H or IDH1‐R132C (*n* = 3). (G) TICs of combined candidate fractions F19 and F20 acquired in positive and negative ion modes. Data are represented as mean ± SEM. *p < 0.05; **p < 0.01; ***p < 0.001; *ns*, not significant. TICs, total ion chromatograms; MS_TIC‐N, negative ion mode total ion chromatogram; MS_TIC‐P, positive ion mode total ion chromatogram; Cps, counts per second; Ctrl, control group; IAA‐yne, iodoacetamide‐alkyne; F, fraction; AG120, ivosidenib; mIDH1, mutant IDH1; *T*_m_, melting temperature; WT, wild type; 2‐HG, 2‐hydroxyglutarate. See also Figure S4.

### Flc is a covalent active molecule derived from *P. methysticum* Forst that targets the mIDH1 protein

We performed virtual covalent docking screening based on the co‐crystal structure of the IDH1 covalent inhibitor IHMT‐IDH1‐053 bound to IDH1‐R132H, as reported by Liang et al. [21]. In this model, IHMT‐IDH1‐053 forms a covalent bond with Cys269 of IDH1‐R132H, thereby inhibiting the production of 2‐HG (Figure 4A). Using Cys269 as the active site, we conducted virtual covalent docking screening of the small molecules identified in Fractions 19 and 20 (Figure 4B, Table S3). Molecules with docking scores below −7 were selected as candidates and individually mapped onto the total ion chromatogram (TIC) plot (Figure 4B,C). Subsequently, Flc was identified through Ccc‐Chip experiments, demonstrating competitive binding against IAA‐yne to both wild‐type and mutant IDH1 proteins (Figure 4D). Docking analysis revealed that Flc forms a covalent bond with Cys269 of IDH1‐R132H (Figure 4E). Comparison of retention times between the Flc standard and the *P. methysticum* Forst. extract showed corresponding chromatographic peaks at 47 min for both (Figure S5). Cellular thermal shift assay (CETSA) experiments further demonstrated that Flc significantly increased the thermal stability of the IDH1‐R132H protein (Figure 4F, Figure S6A). To confirm covalent binding, MALDI‐TOF/TOF analysis was performed on IDH1‐R132H and the IDH1‐R132H‐Flc complex, revealing a significant increase in the molecular weight of the complex (Figure 4G). Finally, LC–MS/MS analysis identified Cys269 as the specific binding site for Flc modification on IDH1‐R132H (Figure 4H).

**Figure 4:**
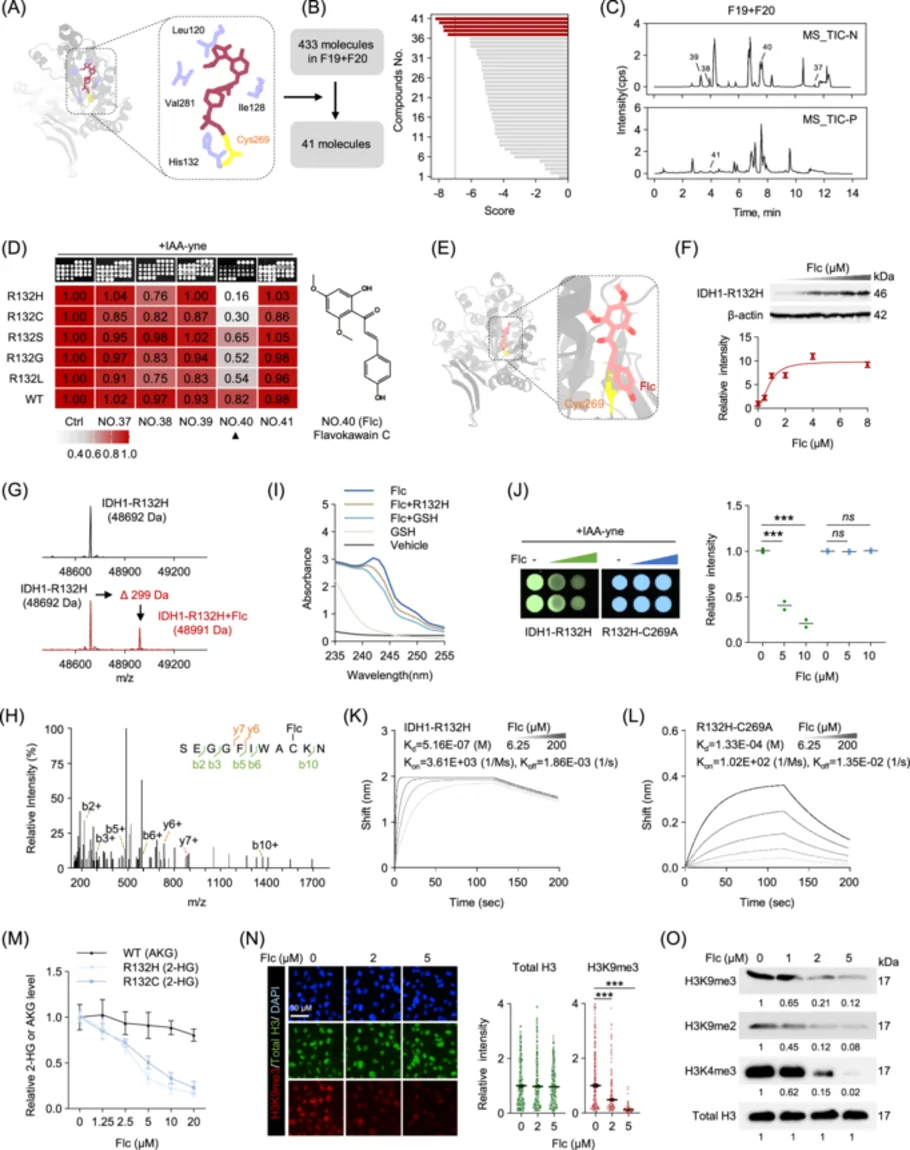
Identification of a covalent IDH1‐R132H inhibitor from *P. methysticum* Forst extracts. (A) Structural model used for covalent virtual screening (PDB ID: 8HB9), showing the binding pocket and interaction between Cys269 and compound IHMT‐IDH1‐053. (B) Covalent virtual screening scores targeting Cys269 of IDH1‐R132H among compounds from fractions F19 and F20. (C) Annotation of the top five candidate molecules in the TIC chromatogram. (D) Ccc‐Chip validation of the top five candidate molecules targeting mIDH1 (*n* = 4). (E) Covalent binding mode of Flc with Cys269 of IDH1‐R132H. (F) CETSA showing the effect of 10 μM Flc on IDH1‐R132H stability in TS603 cell lysates; β‐actin served as a negative control (*n* = 3). (G) Mass spectrometry analysis of IDH1‐R132H treated with PBS or Flc. (H) LC‐MS/MS identification of Flc‐mediated modification of IDH1‐R132H at Cys269. (I) Absorption spectra of 10 μM Flc in the presence or absence of GSH or recombinant IDH1‐R132H protein. (J) Ccc‐Chip analysis of Flc binding to IDH1‐R132H/C269A mutant protein (*n* = 2). (K, L) Biolayer interferometry (BLI) analysis of the binding affinity between Flc and IDH1‐R132H or IDH1‐R132H‐C269A (*n* = 3). (M) Measurement of 2‐HG levels in TS603 and HT1080 cells and AKG levels in HUVECs (*n* = 3). Effects of Flc on histone H3 methylation in TS603 cells assessed by immunofluorescence (N) and Western blotting (O). Scale bars, 50 μm (*n* = 3). Data are represented as mean ± SEM. *p < 0.05; **p < 0.01; ***p < 0.001; *ns*, not significant. AKG, α‐ketoglutarate; CETSA, cellular thermal shift assay; Cps, counts per second; MS_TIC‐N, negative ion mode total ion chromatogram; MS_TIC‐P, positive ion mode total ion chromatogram; Flc, flavokawain C; H3, histone H3; Ctrl, control group; IAA‐yne, iodoacetamide‐alkyne; F, fraction; WT, wild type; *K*_d_, dissociation constant; *K*_on_, association rate constant; *K*_off_, dissociation rate constant; GSH, glutathione; 2‐HG, 2‐hydroxyglutarate. See also Figures S5–S6 and Table S3.

To verify the residues involved in the covalent binding of Flc to the IDH1‐R132H protein, we exploited Flc's characteristic strong absorption between 235 nm and 255 nm, a property known to diminish upon reaction with thiol groups such as those in glutathione [19] (Figure S6B). Consistent with this, we observed a similar reduction in the UV–Vis absorption spectrum of Flc following incubation with IDH1‐R132H (Figure 4I). Next, we assessed Flc's binding capacity to a Cys269‐mutated IDH1‐R132H protein (IDH1‐R132H‐C269A) using a protein microarray. The results demonstrated that the competitive binding between Flc and IAA‐yne was abolished in the mutant protein lacking the cysteine residue at position 269 (Figure 4J). Binding affinity measurements (K_d_) revealed that Flc binds to wild‐type IDH1 (IDH1‐WT) with a K_d_ of 3.92 μM, and exhibits stronger affinities for the mutant proteins IDH1‐R132H and IDH1‐R132C with K_d_ values of 0.516 μM and 0.437 μM, respectively (Figure 4K, Figure S6C,D). Notably, mutation of Cys269 significantly reduced Flc's binding affinity to IDH1‐R132H (*K*_d_ = 133 μM), which confirms that Cys269 is the primary binding site for Flc. (Figure 4L). Analysis of the association rate constant (*K*_on_) and dissociation rate constant (*K*_off_) indicated that Flc binding exhibits rapid association and slow dissociation kinetics, consistent with a “pseudo‐irreversible” interaction characteristic of covalent bond formation (Figure 4K). Furthermore, the reversal of this kinetic profile upon C269A mutation demonstrates that Cys269 is the covalent binding site for Flc on mIDH1 (Figure 4L). Given that Flc also binds to IDH1‐WT, we compared its cytotoxic effects on non‐neoplastic cells expressing wild‐type IDH1 and mIDH1 tumor cells. CCK‐8 assays showed that Flc inhibited TS603 and HT1080 tumor cells comparably to the clinical inhibitor AG120 (Figure S6E). Importantly, at a concentration of 100 μM, Flc exhibited no significant toxicity toward BV2, HUVEC, and ADSC cells, all of which are non‐cancerous and endogenously express IDH1‐WT (Figure S6F). Further investigation of Flc's effects on intracellular metabolites revealed that, at concentrations ranging from 2.5 to 20 μM, Flc significantly suppressed 2‐HG levels in TS603 and HT1080 cells without notably affecting α‐ketoglutarate (AKG) levels in HUVECs (Figure 4M). Additionally, Flc markedly inhibited the methylation levels of histone H3 in TS603 cells (Figure 4N,O). In summary, using the Ccc‐Chip strategy, the natural covalent mIDH1 inhibitor Flc was successfully identified and characterized from *P. methysticum* Forst.

### Flc inhibits 2‐HG in mIDH1 tumors and activates cGAS‐STING‐T‐Cell immunity

BALB/c nude mice bearing TS603‐R132H xenograft tumors received a single intraperitoneal injection of 60 mg/kg Flc, with 40 mg/kg AG120 serving as a positive control (Figure 5A). Subcutaneous tumor samples were collected at specified time points within 2 h post‐administration to measure intratumoral 2‐HG levels. The inhibition of mIDH1 activity was assessed based on the reduction of 2‐HG within the tumors (Figure 5A–C). Results demonstrated that, in this subcutaneous xenograft model, Flc and AG120 suppressed intratumoral 2‐HG levels by approximately 67.1% and 76.8%, respectively, sustained for up to 2 h post‐treatment (Figure 5B,C). Next, the effect of Flc on tumor growth was evaluated after 21 consecutive days of administration in the nude mouse xenograft model (Figure S7A). Flc exhibited a favorable safety profile, as indicated by no significant difference in body weight compared to controls and no observable abnormalities in H&E staining of various tissues (Figure S7B, Figure S8). Although both Flc and AG120 significantly inhibited histone H3 methylation levels in tumor tissues, neither compound demonstrated a significant effect on tumor growth (Figure S7C–H). Previous studies have shown that mIDH1‐driven tumors often evade immune surveillance by excluding cytotoxic CD8^+^ T cells, and mIDH1 inhibitors suppress tumor growth by activating antitumor immunity [22]. However, nude mice are congenitally athymic and thus lack mature T cells, consistent with our immunohistochemical analysis, which revealed no T cell infiltration in the tumor tissues of this model (Figure S9). Therefore, the absence of antitumor efficacy observed with Flc treatment may be attributed to the immunodeficient status of the nude mice, which prevents activation of the tumor immune microenvironment (TIME).

**Figure 5:**
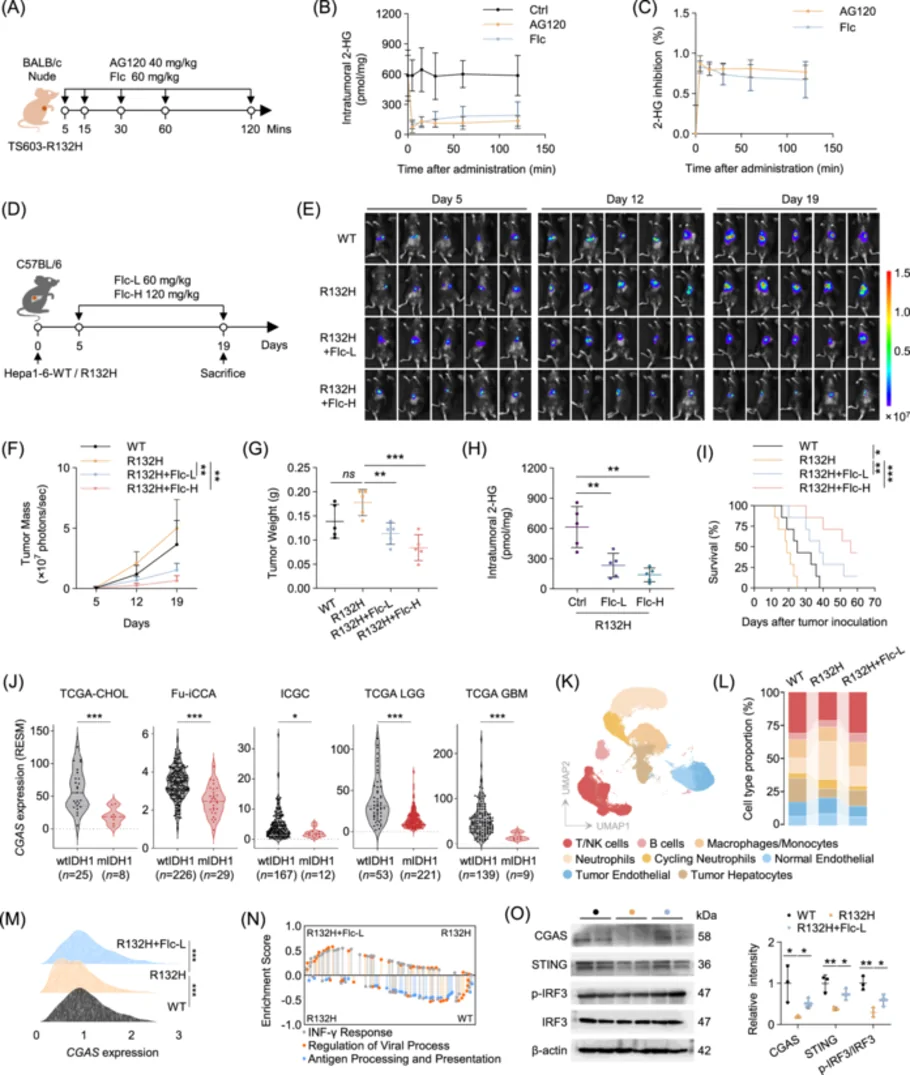
Flc suppresses mIDH1‐driven hepatocellular carcinoma and activates the cGAS signaling pathway. (A) Schematic overview of 2‐HG detection in the TS603 subcutaneous nude mouse model. (B) Tumor 2‐HG concentrations following treatment with Flc or AG120 (*n* = 4). (C) Percentage inhibition of tumor 2‐HG levels following treatment with Flc or AG120 (*n* = 4). (D) Treatment scheme for Flc administration in the orthotopic Hepa1‐6‐R132H hepatocellular carcinoma model. (E‐H) Effects of Flc (60 and 120 mg/kg/day) on tumor growth assessed by bioluminescence imaging (E, F), tumor weight (G), and tumor 2‐HG levels (H) in the orthotopic Hepa1‐6‐R132H model (*n* = 5). (I) Survival analysis of mice treated with Flc in the orthotopic Hepa1‐6‐R132H model (*n* = 7). (J) RNA‐seq analysis of *cGAS* mRNA expression in IDH1‐mutant versus IDH1‐WT tumors from ICC, LGG, and GBM cohorts in the TCGA, Fu‐iCCA, and ICGC datasets. (K) UMAP visualization of single‐cell RNA‐seq data from orthotopic tumors, colored by annotated cell types. (L) Proportional distribution of cell populations across IDH1‐WT, IDH1‐R132H, and IDH1‐R132H + Flc groups. (M) Single‐cell RNA‐seq analysis of *cGAS* expression in tumor cells. (N) Gene set enrichment analysis (GSEA) of differentially expressed genes. (O) Western blot analysis of cGAS, STING, and p‐IRF3/IRF3 in orthotopic Hepa1‐6‐R132H tumors (*n* = 4). Data are represented as mean ± SEM. *p < 0.05; **p < 0.01; ***p < 0.001; *ns*, not significant. Ki67, marker of proliferation Ki‐67; 5‐hmC, 5‐hydroxymethylcytosine; cGAS, cyclic GMP‐AMP synthase; STING, stimulator of interferon genes; p‐IRF3, phosphorylated interferon regulatory factor 3; IRF3, interferon regulatory factor 3; TCGA, the Cancer Genome Atlas; CHOL, cholangiocarcinoma; Fu‐iCCA, Fudan University intrahepatic Cholangiocarcinoma cohort; ICGC, the International Cancer Genome Consortium; LGG, low‐grade glioma; GBM, glioblastoma; IFN‐γ, Interferon‐γ. See also Figures S7–S10.

Furthermore, an IDH1‐R132H orthotopic liver cancer model was established in immunocompetent C57BL/6 mice to evaluate the antitumor efficacy of Flc within a functional immune system (Figure 5D, Figure S10A). The results demonstrated that Flc significantly inhibited the growth of IDH1‐R132H orthotopic tumors, reduced intratumoral 2‐HG production, and prolonged the survival of treated mice (Figure 5E–I). Although no significant difference in tumor growth was observed between IDH1‐R132H and IDH1‐WT tumors within 21 days, survival was markedly shorter in mice bearing IDH1‐R132H tumors compared to those bearing IDH1‐WT tumors (Figure 5I). Previous studies and RNA sequencing datasets from multiple sources have shown that *cGAS* is among the most significantly downregulated genes in various tumor samples from patients with IDH1 mutations, contributing critically to the formation of an immunosuppressive tumor microenvironment [22, 23, 24, 25, 26] (Figure 5J). Consistent with these observations, single‐cell sequencing of IDH1‐R132H orthotopic tumors revealed a significant reduction in the proportion of T/NK cells and markedly decreased *cGAS* expression in tumor cells compared to the IDH1‐WT group, effects that were notably reversed by Flc treatment (Figure 5K–M, Figure S10B). Gene set enrichment analysis (GSEA) of tumor cells showed that, in line with prior findings [22], the IFN‐γ Response, Regulation of Viral Process, and Antigen Processing and Presentation pathways were significantly downregulated in IDH1‐R132H tumor cells relative to IDH1‐WT cells (Figure 5N). However, Flc treatment significantly upregulated the IFN‐γ Response and Regulation of Viral Process pathways in IDH1‐R132H tumor cells (Figure 5N). Furthermore, compared to IDH1‐R132H tumors, both Flc treatment and the IDH1‐WT group showed markedly reduced histone H3 methylation, significantly enhanced cGAS‐STING signaling, and increased CD8^+^ T cell infiltration within tumor tissues (Figure 5O, Figure S10C,D). In support of these results, nude mouse xenograft assays demonstrated that both Flc and AG120 significantly activated the cGAS‐STING pathway in tumor tissues (Figure S7I). Collectively, these findings reveal that IDH1‐R132H tumors exhibit tumor immune suppression, and that Flc can restore T cell‐mediated antitumor immunity via activation of the cGAS‐STING signaling pathway, thereby inhibiting tumor growth.

### Flc suppresses the growth of mIDH1 orthotopic tumors by activating CD8^+^ T cells

Subcluster analysis of T/NK cells revealed significantly lower infiltration of CD8^+^ cytotoxic T cells and higher infiltration of CD8^+^ exhausted T cells in the IDH1‐R132H group compared to the IDH1‐WT group (Figure 6A,B, Figure S11A). Flc treatment increased the proportion of CD8^+^ cytotoxic T cells while reducing the proportion of CD8^+^ exhausted T cells in IDH1‐R132H orthotopic tumors (Figure 6A,B). Moreover, analysis of cytotoxicity and exhaustion scores for CD8^+^ effector T cells demonstrated that Flc significantly enhanced the tumor‐killing capacity of CD8^+^ T cells and alleviated T cell exhaustion in these tumors (Figure 6C,D). Single‐cell RNA sequencing (scRNA‐seq) analysis provides comprehensive insights into intercellular crosstalk. As shown in Figure 6E, T/NK cells exhibited more extensive interactions with other cell types, especially tumor cells, in both the IDH1‐WT and IDH1‐R132H + Flc treatment groups compared to the IDH1‐R132H group alone. These data highlight the critical role of T/NK cells in the cellular communication network during IDH1‐R132H tumor progression and following Flc treatment. We next evaluated dynamic changes in ligand‐receptor (L‐R) pair‐mediated communication between T/NK cells and tumor cells. Notably, L‐R pairs involved in immune cell recruitment and infiltration (*Itgb2‐Icam1, Vcam1*‐(*Itga4+Itgb7*), *Pecam1‐Pecam1*), antigen recognition and T cell activation (*H2‐k1‐Cd8b1, H2‐d1‐Cd8b1*), as well as chemokine signaling and T cell recruitment (*Cxcl9/Cxcl10‐Cxcr3*) showed reduced interaction strength in IDH1‐R132H tumors (Figure 6F). In contrast, Flc treatment restored and enhanced these tumor‐killing‐related L‐R pairs in IDH1‐R132H tumors (Figure 6F). These findings demonstrate that Flc remodels the immunosuppressive TIME into an immunologically active state that is more responsive to immunotherapy, primarily through modulation of CD8^+^ T cell subsets.

**Figure 6:**
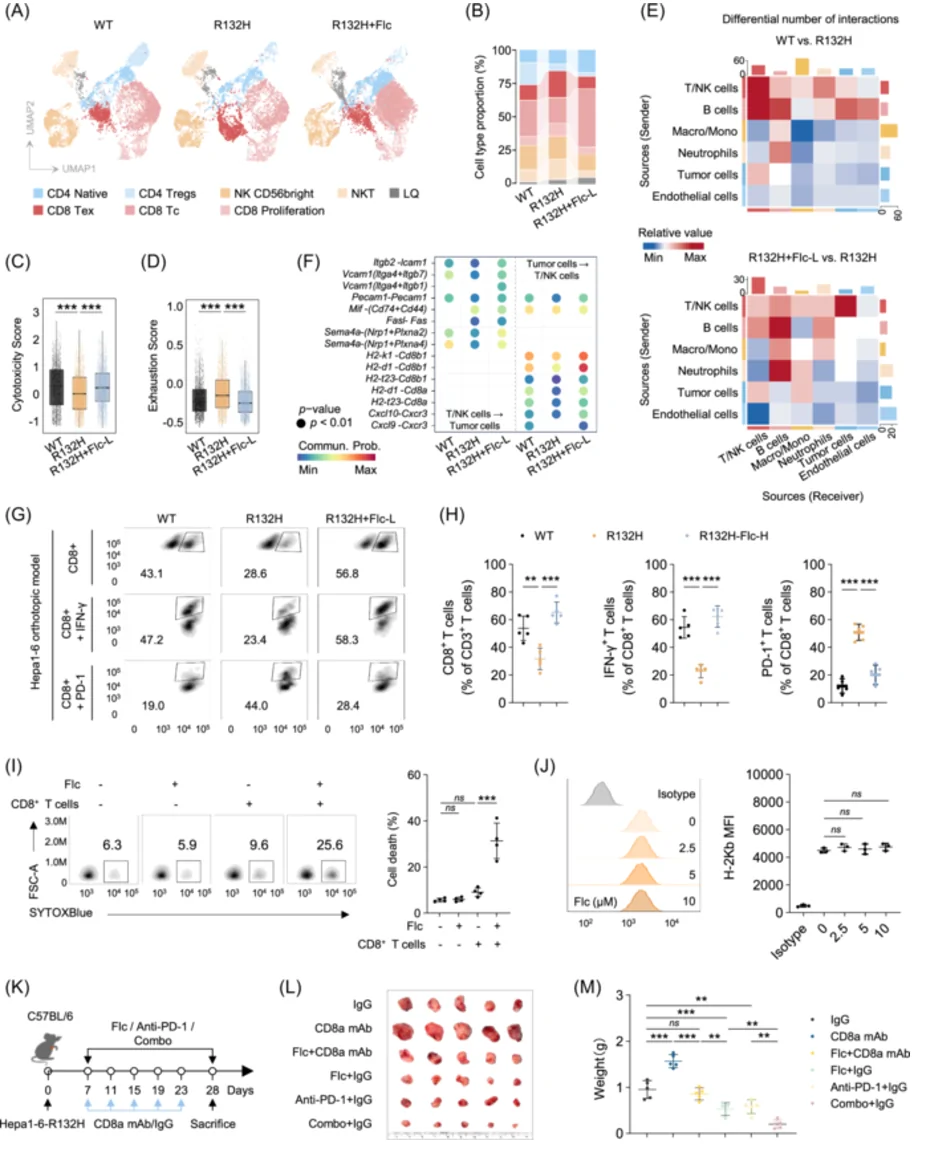
Flc enhances CD8^+^ T‐cell‐mediated antitumor immunity in hepatocellular carcinoma. (A) UMAP visualization of T/NK cell subclusters derived from scRNA‐seq data. (B) Proportional distribution of T/NK cell subpopulations across IDH1‐WT, IDH1‐R132H, and IDH1‐R132H + Flc groups. (C, D) Cytotoxicity and exhaustion scores of CD8^+^ effector T cells under each condition. (E) Heatmaps showing differential ligand‐receptor interaction counts between WT versus R132H and R132H versus R132H + Flc groups. (F) Bubble plot illustrating significant tumor immunity‐related ligand‐receptor interactions among sender‐receiver cell types across the three groups. (G, H) Flow cytometric analysis of CD45^+^CD3^+^ cells from orthotopic tumors showing infiltration of total CD8^+^ T cells (G) and IFN‐γ^+^ and PD‐1^+^ CD8^+^ T cells (H) following Flc treatment (*n* = 5). (I) Effect of 10 μM Flc on antigen‐specific cytotoxicity of CD8^+^ T cells co‐cultured with Hepa1‐6‐R132H cells, assessed by flow cytometry (*n* = 4). (J) MFI of H‐2Kb on Hepa1‐6‐R132H cells following Flc treatment (*n* = 3). (K‐M) Experimental design (K), representative images (L), and tumor weights (M) of subcutaneous tumor models treated with anti‐CD8α mAb (100 μg/mouse, 3 times/week, five injections in total), Flc (60 mg/kg, daily), and anti‐PD‐1 antibody (200 μg/mouse, 3 times/week, five injections in total) (*n* = 5). Data are represented as mean ± SEM. *p < 0.05; **p < 0.01; ***p < 0.001; *ns*, not significant. CD4 Native, native CD4^+^ T cells; CD4 Tregs, CD4^+^ regulatory T cells; CD8 Proliferation, proliferating CD8^+^ T cells; CD8 Tc, cytotoxic CD8^+^ T cells; CD8 Tex, exhausted CD8^+^ T cells; NK CD56bright; NKT, natural killer T cells; LQ, low‐quality cells; MFI, mean fluorescence intensity; IFN‐γ, Interferon‐γ; PD‐1, programmed cell death protein 1; H‐2Kb, H‐2 class I histocompatibility antigen; IgG, immunoglobulin G isotype control; Combo, combination. See also Figure S11.

Flow cytometric analysis of tumor tissues revealed that, compared to IDH1‐WT tumors, IDH1‐R132H tumors exhibited a significant decrease in the proportions of tumor‐infiltrating CD8^+^ T cells and IFN‐γ^+^ CD8^+^ T cells, alongside a significant increase in the proportion of PD‐1^+^ CD8^+^ T cells. Flc administration notably reversed these changes by significantly increasing the proportions of tumor‐infiltrating CD8^+^ and IFN‐γ^+^ CD8^+^ T cells while decreasing the proportion of PD‐1^+^ CD8^+^ T cells within IDH1‐R132H tumors (Figure 6I,J). To further validate Flc's enhancement of CD8^+^ T cell‐mediated cytotoxicity, we established a co‐culture system of IDH1‐R132H Hepa1‐6 cells and CD8^+^ T cells (Figure S11C). CD8^+^ T cells pretreated with conditioned medium from Flc‐incubated IDH1‐R132H Hepa1‐6 cells exhibited augmented antitumor activity (Figure S11D, Figure 6I). Intercellular crosstalk analysis highlighted enhanced communication of the *H2‐k1‐Cd8b1* L‐R pair, implicated in antigen recognition and T cell activation. However, flow cytometry indicated that Flc did not alter H‐2Kb‐mediated antigen presentation by IDH1‐R132H Hepa1‐6 cells, suggesting that the increased *H2‐k1‐Cd8b1* interaction results primarily from enhanced CD8^+^ T cell infiltration (Figure 6J). To further confirm the critical role of CD8^+^ T cells in Flc‐mediated tumor immunoregulation, a rescue experiment was conducted using a CD8a monoclonal antibody (mAb) (Figure 6K). Neutralization of CD8^+^ T cells significantly attenuated the inhibitory effect of Flc on tumor growth (Figure 6L,M), verifying that Flc suppresses mIDH1 hepatocellular carcinoma progression at least in part through CD8^+^ T cell‐dependent mechanisms. Importantly, combining Flc with a PD‐1 antibody produced a synergistic inhibitory effect on mIDH1 tumors (Figure 6L,M), suggesting that co‐targeting mIDH1 and immune checkpoints to modulate the immunosuppressive tumor microenvironment may enhance therapeutic efficacy. In summary, our in vivo experiments and scRNA‐seq analyses collectively demonstrate that Flc possesses immune‐activating properties, likely mediated via CD8^+^ T cell interactions, and exerts a positive regulatory effect on the TIME.

## DISCUSSION

In this study, we developed an integrated screening platform, the Ccc‐Chip, which synergistically combines protein microarray technology with bioorthogonal click chemistry to directly screen complex natural extracts. This approach enabled high‐throughput screening of 110 medicinal plant extracts, leading to the identification of Flc from *P. methysticum* Forst. as a covalent inhibitor targeting mutant IDH1. Serving as the core decision‐making step, the Ccc‐Chip platform specifically confirmed the covalent binding nature of Flc's interaction with its target. Mechanistic investigations demonstrated that Flc covalently binds to Cys269 of mutant IDH1, effectively suppressing oncometabolite 2‐HG production and subsequently reactivating CD8^+^ T cell‐mediated antitumor immunity within the tumor microenvironment.

We integrated DSF as an initial high‐throughput screening tool due to its simplicity, rapid turnaround, and low cost—requiring only a qPCR instrument and minimal protein input. This low‐barrier approach enables broad accessibility across molecular biology laboratories, allowing efficient primary screening of hundreds of complex natural extracts with varying compositions and concentrations. Skipping this step and directly applying Ccc‐Chip to all samples, though technically feasible, would incur higher costs (probes, chips) and time investments in large‐scale initial screening. Regarding threshold selection for DSF screening, we rigorously validated a ΔT_m_ threshold (1.5°C) using five positive control molecules (AG881, AG120, FT2102, Scu, and Cpd13)—representing covalent/non‐covalent mIDH1‐targeting ligands—and the Scu‐IDH1‐C297S complex as a negative control [19, 27]. Through strict dual validation with these positive and negative controls, this threshold effectively balances sensitivity and specificity, aiming to reduce risks of false positives (non‐specific binding and background noise) and false negatives (weak covalent binders). However, DSF's utility is tempered by inherent limitations: it cannot distinguish covalent from non‐covalent binders. DSF serves as a broad, high‐throughput filter for potential binders, while the subsequent Ccc‐Chip assay provides a highly specific confirmation of covalent engagement at cysteine residues. This sequential application of orthogonal methods enhances the overall robustness of hit identification by ensuring that only extracts capable of both modulating protein stability and forming covalent bonds with the target cysteine are advanced. Although this strategy involves a compromise between throughput and mechanistic specificity, the Ccc‐Chip platform ultimately serves as the definitive confirmation tool, exemplified by the successful identification of Flc.

The current implementation of the Ccc‐Chip platform employs a cysteine‐reactive iodoacetamide probe (IAA‐yne), which reliably identifies cysteine‐targeting covalent ligands. Although the platform's modular design could theoretically accommodate probes targeting other nucleophilic amino acids, such as lysine‐directed sulfotetrafluorophenyl ester probes, tyrosine‐specific sulfur(VI)‐fluoride exchange probes, or photoactivatable reagents for aspartate/glutamate residues, experimental validation of these expanded capabilities remains to be systematically explored [28, 29, 30]. Expanding the probe repertoire will be essential to establishing Ccc‐Chip as a truly universal platform for covalent drug discovery. The platform's adaptability is underscored by its compatibility with target‐directed probes bearing bioorthogonal handles, paving the way for future high‐throughput screening campaigns tailored to diverse protein targets.

The chalcone scaffold of Flc, characterized by its highly reactive Michael acceptor moiety, warrants attention regarding potential pan‐assay interference compounds (PAINS) concerns and target specificity [31]. Although genetic knockout validation of mIDH1 is absent in this study, multiple lines of evidence strongly support mIDH1 as a critical functional target of Flc. This includes its high selective inhibitory potency against mIDH1, its characteristic fast‐association/slow‐dissociation binding kinetics critically dependent on the Cys269 residue, and its effective reduction of the oncometabolite 2‐HG coupled with activation of relevant antitumor immune pathways in cellular models. Collectively, while contributions from secondary mechanisms cannot be entirely ruled out, our data indicate that inhibition of mIDH1 represents a primary pathway mediating the antitumor effects of Flc. Regarding the pharmacological profile of the identified compound, Flc exhibits preferential binding affinity for mutant IDH1 variants (IDH1‐R132H and IDH1‐R132C) over the wild‐type enzyme. However, the selectivity window, approximately 8 to 9‐fold, indicates room for further optimization through structural modifications. Although Flc causes modest inhibition of IDH1‐WT enzymatic activity (8.9% at 5 μM), no significant cytotoxicity was observed in multiple IDH1‐WT‐expressing normal cell lines (BV2, HUVEC, and ADSCs) even at concentrations up to 100 μM. This suggests that within the efficacious concentration range (IC50~0.5 μM for mutant IDH1), the modest wild‐type inhibition is unlikely to result in appreciable on‐target toxicity, supporting a potentially acceptable therapeutic window. Notably, Flc's inhibitory potency against mutant IDH1 is comparable to that of the clinically approved agent AG‐120, and its favorable safety profile underscores its promise as a lead candidate for further development. In vivo data demonstrate that Flc synergizes with anti‐PD‐1 antibody therapy, enhancing antitumor efficacy. This finding suggests that while Flc monotherapy is effective, combination strategies with immune checkpoint inhibitors may yield superior therapeutic outcomes. Such an approach aligns with current cancer immunotherapy paradigms, which emphasize targeting multiple pathways to overcome tumor immunosuppression [32]. Given that mIDH1‐mutant tumors often exhibit an immune‐cold phenotype, Flc's ability to reduce 2‐HG levels and activate the *cGAS‐STING* pathway and CD8^+^ T cell function may help transform these tumors into immunologically active environments more responsive to PD‐1/PD‐L1 blockade. Therefore, although Flc demonstrates promising biological activity and safety as a single agent in preclinical models, its clinical development may be most impactful as part of combination therapies with PD‐1/PD‐L1 inhibitors. This strategy holds significant potential to improve treatment outcomes for patients with mIDH1‐mutant cancers.

In summary, the Ccc‐Chip platform offers a robust and versatile framework for discovering covalent bioactive molecules from complex natural product sources. The identification and mechanistic validation of Flc not only highlight a promising therapeutic candidate for targeting mIDH1‐driven cancers but also demonstrate the effective integration of natural product chemistry with functional proteomics. This methodology lays a strong foundation for bridging natural product discovery and targeted drug development. Future studies will aim to expand the chemical diversity of probes compatible with the Ccc‐Chip platform, adapt the approach for challenging target classes such as membrane proteins, and undertake detailed structure‐activity relationship investigations to optimize the pharmacological profile of the Flc scaffold.

## CONCLUSION

This study innovatively establishes an integrated screening platform, Ccc‐Chip, which combines protein microarray technology with bioorthogonal click chemistry to enable direct, high‐throughput discovery of natural covalent bioactive molecules from medicinal plants. The platform's versatility lies in its customizable immobilization of diverse target proteins, spanning key signaling and metabolic proteins implicated in various diseases, allowing for multi‐target screening. Additionally, the cysteine‐targeting IAA‐yne probe can be readily replaced with other site‐specific covalent probes tailored to the active site features of target proteins. Applying this platform to 110 medicinal plant extracts led to the identification of Flc, a novel covalent inhibitor of mutant IDH1 derived from *P. methysticum* Forst., which effectively reduces intratumoral 2‐HG levels and enhances CD8^+^ T cell‐mediated antitumor immunity. Overall, the Ccc‐Chip platform offers a robust framework for natural covalent drug discovery, bridging natural product chemistry, chemical biology, and pharmacology to advance targeted covalent therapeutics.

## METHODS

### Cell line and mice

Experiments were conducted in TS603 (Bluefcell, BFN608006424, RRID: CVCL_A5HW), HT1080 (Servicebio, STCC12701P, RRID: CVCL_XZ56), Hepa1‐6‐Luc (Servicebio, STCC00016, RRID: CVCL_RA19), HEK293T cells (BOSTER, CX0122, RRID: CVCL_0063), Human ADSC cells (Saliai, RAD0001530401), PUMC‐HUVEC‐T1 cells (Procell, CL‐0675), and BV2 cells (China Center for Type Culture Collection, SCSP‐5208, RRID: CVCL_0182). TS603, Hepa1‐6‐Luc, BV2, and HEK293T cells were cultured in DMEM (XP Biomed, C3101) supplemented with 10% FBS (Dogesce, DGF8283). HT1080 cells were cultured in MEM (XP Biomed, C3050) supplemented with 10% FBS. ADSC were cultured in OptiVitro® MSC Expansion Medium XF (ExCell Bio, ME000‐N023). PUMC‐HUVEC‐T1 cells were cultured in HUVEC Cell Complete Medium (Procell, CM‐0675). All cells underwent comprehensive quality control assessments, including viability evaluation, microbial contamination detection, specific human‐derived virus screening, cell surface antigen profiling, and differentiation capacity validation, with all results strictly complying with the standards of the International Society for Cellular Therapy (ISCT). Cells were authenticated through STR profiling and regularly tested for mycoplasma using PCR Mycoplasma Detection Kit (Yeasen, 40614ES10). All cells were routinely maintained at 37°C in humidified air containing 5% CO_2_.

Male BALB/c nude mice (4–5 weeks old) and male BALB/c mice (3–4 weeks old) were purchased from SiPeiFu Biotechnology. Mice were maintained under specific pathogen‐free conditions with a 12‐h light/12‐h dark cycle. All care and treatment of the experimental animals were in strict accordance with the guidelines of the Association for Assessment and Accreditation of Laboratory Animal Care approved by the Institutional Animal Care and Use Committee of the Medical Experiment Center, China Academy of Chinese Medical Sciences.

### Compounds preparation

All plant extracts of medicinal plants were purchased from Mufan Biotechnology and Risun Bio‐tech, and dissolved in 80% ethanol (MACKLIN) as stock solutions at 200 μg/mL and stored at −20°C. Flavokawain C (TargetMol, T5753), Apigenin‐7‐O‐glucuronide (Aladdin, A423058), Mangiferolic acid (Chemfaces, CFN97742), Gossypol (TargetMol, T6518), 1,3‐Dicaffeoylquinic acid (Selleckchem, S9512), AG120 (MCE, HY‐18767), AG881 (MCE, HY‐104042), FT2102 (Selleckchem, S8949), Scu (Nature Standard, ST03110120), and Cpd13(MCE, HY‐128661) were dissolved in dimethyl sulfoxide (DMSO) (Sigma) as stock solutions at 100 mM and stored at −20°C. All stock solutions were freshly diluted with medium to their final concentrations before each *in vitro* experiment. The final DMSO concentration did not exceed 0.1%.

### Protein expression and purification

Target cDNA of AKT1, P50, KRAS^G12C^, IDH1‐WT, IDH1‐R132H, IDH1‐R132C, IDH1‐R132G, IDH1‐R132S, IDH1‐R132L was cloned and inserted into the NdeI/Xho I sites of the pET‐28a^+^ vector. The recombinant plasmid was transformed into *E. coli* BL21 (DE3) cells (Vazyme Biotech, C504), which were subsequently cultured in 300 mL of Luria–Bertani (LB) medium at 37°C until the absorbance at OD600 was 0.4–0.6. The cells were subsequently induced with 0.4 mmol/L isopropyl‐Dthiogalactopyranoside (IPTG) for 6 h at 16°C. To obtain the nondenatured protein, cell debris was removed by centrifugation, and the supernatant was loaded onto preequilibrated Ni‐NTA resin (Beyotime, P2218). Proteins were eluted using 200 mM imidazole. Then, the imidazole solution was replaced with a PBS solution by ultrafiltration. Finally, the protein concentration was measured using a BCA kit (Solarbio, PC0020), and the final protein was concentrated to 10 mg/mL and stored at −80°C.

### Generation of IDH1‐R132H overexpression cell lines

The IDH1‐R132H expression plasmids were designed and synthesized by Applied Biological Materials Inc. (Abm). All plasmids were purified with the endotoxin‐free plasmid midi kit (Aidlab,160112) to achieve an A260/280 ratio of 1.8–2.0. For 293T cell packaging, cells are cultured at 80–90% confluence in 10 cm dishes with DMEM/10% FBS, transfected with the transfer vector, packaging plasmids, and envelope plasmid using Lentiviral Packaging Kit (Biorigin, BN80101), followed by medium replacement with fresh DMEM/10% FBS at 12 h post‐transfection. Virus supernatants are collected at 48 h and 72 h, and concentrated via PEG8000 (Beyotime, ST483) precipitation or ultracentrifugation (20,000 × *g*, 2 h). Hepa1‐6 cells were infected with the concentrated virus in the presence of 2 μg/mL polybrene (Beyotime, ST1380), incubated at 37°C for 6 h, and maintained in DMEM Medium thereafter. Stable cell lines are selected with antibiotic at 48 h post‐infection, and target protein expression is confirmed by Western blot analysis at 72 h.

### Preparation and application of the protein chip

The recombinant protein was dissolved in phosphate‐buffered saline (PBS) and then mixed with 50% glycerol at a ratio of 1:1 to prepare a protein printing solution with a final protein concentration of 0.5 μg/μL. BSA‐Cy3 (Bioss, bs‐0292P) and 5% BSA were mixed with 50% glycerol at a ratio of 1:1 to prepare a control printing solution. Fifteen microliters of the prepared printing solution was placed in a 384‐well plate. Preprinted adhesion microscope slides (Citotest) and polymer three‐dimensional substrates (CapitalBio) were mounted on a PersonalArrayer™ 16 contact printing instrument (CapitalBio), and the program was set to print 40 replicates; the printing process was repeated three times. After printing, the protein chip was placed in a 37°C incubator to dry overnight. The prepared protein chip was sealed with a 5% BSA solution at room temperature for 30 min. After blocking, the chip was washed with PBST three times for 5 min each time. The mixture was centrifuged at 1000 rpm for 1 min to spin dry the liquid in the protein chip and stored at 4°C for later use. The signal‐to‐noise ratio (SNR) of the protein chip signal was calculated using the following formula, where SNR is determined by the difference between the mean gray value of the signal region and that of the background region, normalized by the standard deviation of the background region.

### The competitive in‐gel fluorescence labeling of compounds

The recombinant proteins were incubated with different concentrations of compounds at 37°C for 2 h. After treatment with different concentrations of compounds, the recombinant proteins were labeled with the cysteine‐specific probe IAA‐yne (IAA incorporated with an alkyne moiety) for 1 h in a shaker (800 rpm, 37°C). Next, click buffer containing butyltrichloroacetimidate (TBTA), tris(2‐carboxyethyl) phosphine (TCEP), CuSO4, and TAMRA‐azide was added to each sample, which was subsequently incubated in a shaker at 37°C for 2 h. One milliliter of acetone was added to the mixtures to precipitate the labeled proteins at −80°C for 30 min. The supernatant was discarded by centrifugation for 10 min at 20,000 × *g*, and the acetone was evaporated. The samples were dissolved in 100 μL of 1× loading buffer, denatured at 95°C for 10 min, and finally separated by 10% SDS‐PAGE. An Amersham Image Quant 800 (GE Healthcare Life Sciences) was used to capture fluorescence gel images, and Coomassie brilliant blue (CBB) was used to visualize the total proteins.

### The competitive protein chip fluorescence labeling of compounds

The protein chip was incubated with different concentrations of compounds at 37°C for 2 h. After treatment with different compounds, the protein chip was labeled with 10 μM IAA‐yne at 37°C for 1 h. Next, click buffer containing TBTA, TCEP, CuSO4, and TAMRA‐azide was added to each sample, which was subsequently incubated at 37°C for 2 h. Then, the chip was washed with PBST three times for 5 min each time. The mixture was centrifuged at 1000 rpm for 1 min to spin‐dry the liquid in the protein chip. Using Agilent G2565CA Microarray Scanner (Agilent), set the resolution to 10 μm and scan the protein chip in red and green light modes. The protein chip image was read by GenePix™ Pro v6.0 software to obtain the original fluorescence data.

### DSF

DSF method was performed using an Applied Biosystems StepOnePlus Real‐Time PCR System (Thermo Fisher Scientific). The 10 μL recombined proteins (0.15 μg/μL) were mixed with 2 μL compounds solution (10 μg/mL) or 2 μL PBS in a reaction mixture containing 5 μL Protein Thermal Shift buffer and 2.5 μL Protein Thermal Shift dye (Thermo Fisher Scientific, 4461146). Transfer the prepared reaction mixture to a 0.1 mL multi‐strip PCR tube. Then centrifuge to ensure that the solution was at the bottom of PCR tube and then covered with Microamp optical adhesive film. Each melt curve was programmed as follows: 25°C for 2 min, followed by a 1°C increase per min from 25°C to 95°C, and finally 95°C for 2 min. Arbitrary fluorescence was plotted as a function of temperature. The *T*_m_ is defined as the temperature at which fluorescence is highest, corresponding to the maximum amount of dye bound to exposed regions during thermal denaturation. No significant background fluorescence was observed in the absence of protein.

### Cell viability

Cells were plated in 96‐well plates and treated with the indicated compounds. After 24 h of incubation, the cells were incubated with CCK‐8 reagent (MedChemExpress) for another 2 h. Then, the absorbance at 450 nm was determined spectrophotometrically on a Synergy2 multimode microplate reader (BioTek). Cell proliferation is expressed as a percentage relative to that of the control group after subtraction of the background signal.

### Detection of AKG and 2‐HG levels

For the cell samples, the cells were resuspended in PBS, subjected to ultrasonic disruption on ice, and then centrifuged at 8000 × *g* for 10 min at 4°C; the supernatant was collected and placed on ice for analysis. For tissue samples, fresh tissue was homogenized in PBS on ice and then centrifuged at 8000 × *g* for 10 min at 4°C; the supernatant was collected and placed on ice for testing. The AKG concentration was assessed using an α‐ketoglutarate detection kit (Shanghai Bohu, PH19358) according to the manufacturer's instructions. The 2HG concentration was assessed using a D‐2‐Hydroxyglutarate Assay Kit (Sigma, MAK320) according to the manufacturer's instructions.

### CETSA

TS603 cells were treated with Flc (20 μM) or DMSO for 12 h, aliquoted, and heated at different temperatures (37°C and 77°C) for 4 min. After cooling to room temperature, soluble proteins were collected by centrifugation at 20000 × *g* for 5 min at 4°C and then detected by Western blotting.

### Evaluation of the binding ratio of Flc to IDH1‐R132H using MALDI TOF/TOF

Recombinant IDH1‐R132H was incubated with either Flc (1 mM) or DMSO (control) at 37°C for 2 h. After incubation, unbounded Flc and salt were removed by eluting the mixture using C4 desalination column (Thermo Fisher Scientific). The molecular weight of the two products was then measured using MALDI‐TOF/TOF, the results analyzed and the binding ratio of Flc to IDH1‐R132H calculated.

### Identification of binding sites by LC‐MS/MS

Binding sites were identified by LC–MS/MS using an Orbitrap Fusion Lumos mass spectrometer (Thermo Fisher Scientific). Then, 50 μg of IDH1‐R132H was mixed with 1 mM Flc and incubated for 2 h in 200 μL of PBS. Then, 5 mM dithiothreitol was added for 60 min, and 20 mM 3‐indoleacetic acid was added to the mixed solution for alkylation. To remove excess drugs and reagents, a methanol/chloroform system was chosen to precipitate proteins. Next, trypsin (1:100 enzyme/protein ratio) (Promega) was used to cleave the protease into peptides at 37°C overnight. After desalting the C18 column, 0.1% formic acid was used to dissolve the peptides, which were subsequently prepared for MS detection.

### BLI

The recombinant protein was labeled with a twofold molar amount of biotin reagent. Unconjugated biotin was removed by ultrafiltration. The binding affinities of the compounds for recombinant proteins were determined by a biolayer interferometry assay using Gator Plus (Gator Bio). SMAP biosensor tips (Gator Bio, 160011) were used to immobilize the biotin‐labeled proteins after prewetting with kinetic buffer (PBS, 0.05% BSA, 0.01% Tween 20). The equilibrated SMAP biosensors were loaded with recombinant proteins. Background binding controls used a duplicate set of sensors that were incubated in a buffer without proteins. All assays were performed by a standard protocol in 96‐well plates with a total volume of 250 μL/well. All the data were analyzed by Gator Bio data analysis software. The equilibrium *K*_d_ values were calculated from the ratio of *K*_off_ to *K*_on_.

### UV–visible absorption spectroscopy

The UV–visible absorption spectra of Flc in PBS with 10% DMSO were measured at 200–400 nm by a Synergy2 multimode microplate reader (BioTek). Flc was incubated with or without the indicated proteins, and the absorbance spectra were recorded.

### Western blotting

Whole‐cell lysates were prepared using RIPA lysis buffer (Epizyme) supplemented with complete protease inhibitor cocktail (Selleck), homogenized, and centrifuged at 12000× *g* for 10 min at 4°C. The protein concentration of the cell lysates was determined by the BCA protein assay reagent (Solarbio). The cell lysates were incubated in SDS–PAGE sample loading buffer at 95°C for 10 min, separated by 8%–12% SDS‐PAGE, and transferred to PVDF membranes (Millipore). The membranes were blocked with 5% skim milk (Beyotime, P0216) at 25°C for 30 min and then incubated with the indicated primary antibodies against IDH1 (Proteintech, 12332‐1‐AP, RRID: AB_2123159), Histone H3 (Sino Biological, 100005‐MM01, RRID: AB_2860034), H3K9me3 (ABclonal, A22295, RRID: AB_3066640), H3K9me2 (Invitrogen, 49‐1007, RRID: AB_2533858), H3K4me3 (ABclonal, A22146, RRID: AB_3066542), CGAS (Proteintech, 29958‐1‐AP, RRID: AB_2935491), STING (Proteintech, 80165‐1‐RR, RRID: AB_2918870), p‐IRF3 (ABclonal, AP0995, RRID: AB_2863887), IRF3 (ABclonal, A2172, RRID: AB_476695), or β‐actin (ABclonal, AC026, RRID: AB_2768234) overnight at 4°C. Subsequently, the membranes were incubated with horseradish peroxidase (HRP)‐conjugated anti‐rabbit or anti‐mouse IgG secondary antibodies (Beyotime) for 1 h at room temperature. SuperKine^TM^ hypersensitive ECL luminescent solution (Abbkine, BMU102) was used for the detection of the proteins of interest. The membranes were analyzed by an SH‐520 Gel Imaging Analysis System (Shenhua Bio) and quantified by ImageJ software.

### Immunofluorescence assay

The cells were seeded onto glass coverslips (Biosharp), treated with the indicated compounds for 48 h, and fixed in 4% paraformaldehyde (Beyotime, P0099) for 5 min. After being washed with PBS three times, the cells were permeabilized with Triton X‐100 (Beyotime, P0096) for 10 min, blocked with 5% BSA (ABclonal, RM02802) for 60 min at room temperature, and probed with the indicated primary antibodies against Histone H3 (Sino Biological, 100005‐MM01, RRID: AB_2860034) and H3K9me3 (ABclonal, A22295, RRID: AB_3066640) for 2 h at room temperature. Then, the cells were exposed to Alexa Fluor 594‐labeled anti‐rabbit or FITC‐labeled anti‐mouse secondary antibodies (Bioss) and stained with 4,6‐diamidino‐2‐phenylindole (DAPI) (Beyotime, P0131). Image acquisition was achieved using an EVOS M7000 intelligent imaging system (Thermo Fisher Scientific) and a Cytation 5 Multi‐Functional Cell Imaging Microplate Detection System (Agilent).

### Immunohistochemical staining

All tissue chips and sections were deparaffinized in xylene and rehydrated in water through descending‐graded alcohols. Each block had a section for hematoxylin and eosin (H&E) staining. Heat‐induced antigen retrieval was achieved by incubation in 0.01 M citrate buffer at 90°C for 20 min. Attenuation of endogenous peroxidases was achieved by incubation in 3% hydrogen peroxide. The sections were blocked in PBS containing 10% normal goat serum and 0.3% Triton X‐100 for 60 min; labeled with primary antibodies against Ki67 (Proteintech, 27309‐1‐AP, RRID: AB_2756525), 5‐hmC (Activemotif, 39092, RRID: AB_2630381), H3K9me3 (ABclonal, A22295, RRID: AB_3066640), CGAS (Proteintech, 29958‐1‐AP, RRID: AB_2935491), and CD8 (Proteintech, 66868‐1‐Ig, RRID: AB_2882205) overnight at 4°C; and incubated with the corresponding goat secondary antibodies for 1 h at room temperature. Detection was accomplished using a 3,3'‐diaminobenzidine (DAB) substrate kit (Solarbio). The slices were then stained with hematoxylin and examined using a CKX53 microscope (Olympus).

### Obtaining semi‐prepared components of *Piper methysticum* Forst extract

A 1.5 mg aliquot of *Piper methysticum*Forst extract was weighed and extracted with eight volumes of 80% ethanol (MACKLIN) using ultrasonication for 30 min, followed by filtration. The mixture was centrifuged at 12,000 rpm for 10 min to obtain a clear supernatant, which was aliquoted and dried under a nitrogen stream. For semi‐preparative separation, the dried material was reconstituted in 70% methanol (Fisher) to a concentration of 100 mg/mL (based on crude drug weight) and filtered through a 0.22 μm membrane. A 200 μL sample was injected into an HPLC system (Beijing Tong Heng Innovation Technology) equipped with a chromatographic column (10 mm × 250 mm, FLB‐10250‐5μm‐100A). The mobile phase consisted of acetonitrile (Fisher) and 0.1% aqueous formic acid (MACKLIN) with the following gradient program: 10–60% acetonitrile (0–10 min), 60–70% (10–35 min), 70–95% (35–60 min), and 95–100% (60–70 min), at a flow rate of 1 mL/min. Based on the elution profile, 35 consecutive fractions were collected at 1‐min intervals from 10 to 80 min, covering a period starting 5 min before the first absorption peak and ending 5 min after the last absorption peak to ensure comprehensive coverage of the chemical constituents. The collected fractions were dried under nitrogen, and each residue was redissolved in 10 μL of DMSO. A 1 μL aliquot of each fraction was used for DSF and competitive protein chip assays (20 μL total volume).

### UPLC‐MS/MS identification of small molecules in active components

Sample extraction was performed by adding 100 μL of sample into correspondingly labeled 1.5 mL centrifuge tubes, followed by the addition of 100 μL of 70% methanol containing an internal standard extraction solution, which was prepared by first dissolving 1 mg of the standard in 1 mL of 70% methanol aqueous solution to prepare a 1000 μg/mL stock solution, which was then further diluted with 70% methanol to prepare a 250 μg/mL internal standard working solution. The mixture was vortexed for 15 min and then centrifuged at 12,000 r/min for 3 min at 4°C. The supernatant was collected, filtered through a 0.22 μm microporous membrane, and stored in injection vials for LC‐MS/MS analysis. The data acquisition system consisted of an UPLC system (ExionLC™ AD) coupled with a MS/MS system. The liquid chromatography conditions were as follows: column: Agilent SB‐C18 (1.8 μm, 2.1 mm × 100 mm); mobile phase: A, ultrapure water with 0.1% formic acid, and B, acetonitrile with 0.1% formic acid; elution gradient: 0.00 min, 5% B, increased linearly to 95% B over 9.00 min, maintained at 95% B until 10.00 min, then decreased to 5% B from 10 to 11 min, and equilibrated at 5% B until 14 min; flow rate: 0.35 mL/min; column temperature: 40°C; injection volume: 2 μL. The MS conditions were as follows: electrospray ionization (ESI) source temperature: 500°C; ion spray voltage (IS): 5500 V (positive ion mode)/−4500 V (negative ion mode); ion source gas I (GSI), gas II (GSII), and curtain gas (CUR) set at 50, 60, and 25 psi, respectively; collision‐induced ionization parameter set to high. QQQ scans were performed using multiple reaction monitoring (MRM) mode with collision gas (nitrogen) set to medium. Specific MRM ion pairs were monitored for each metabolite based on their respective elution periods. Metabolite identification was performed using the MWDB (MetWare Database) established by Metware Biotechnology, based on MS/MS spectral information. Metabolite quantification was carried out using MRM mode with triple quadrupole MS.

### Virtual covalent screening

First, the crystal structure of IDH1‐R132H (PDB: 8HB9) was retrieved from the Protein Data Bank (PDB) and optimized using the Schrödinger Protein Preparation Wizard: hydrogen atoms were added automatically, missing residues/side chains were repaired at pH 7.0 to ensure Cys269 maintained a reduced thiol (‐SH), and the structure was refined with the OPLS4 force field; irrelevant crystal waters were removed, yielding the preprocessed protein. Next, LC‐MS/MS data of fractions 19 and 20 were parsed, and small molecule structures were identified via database searching against MassBank and HMDB combined with fragment ion analysis, then converted to SMILES/SDF formats. The small molecules were preprocessed using LigPrep: up to 50 3D conformations were generated per molecule via the ETKDG v3 method, and their ionization states and charges at physiological pH were calculated using the OPLS4 force field, producing a normalized small molecule library. Finally, covalent docking was performed using the Glide Covalent Docking module in Schrödinger: Cys269's sulfur atom (S) was defined as the nucleophilic center with the Michael Addition reaction type selected; a 10 × 10 × 10 Å docking grid centered on Cys269 was generated, retaining the top 5 docking poses per small molecule; batch processing via Maestro or Tcl scripting was employed to run docking using the preprocessed protein and small molecule library, outputting the docking results.

### In vivo antitumor efficacy evaluation

For the subcutaneous tumor model, human and mouse hepatocellular carcinoma cells were separately injected subcutaneously into the right flank of BALB/c mice (1 × 10^6^ cells/100 μL PBS) and C57BL/6 mice (2 × 10^6^ cells/100 μL PBS) to generate subcutaneous tumor models. Tumor growth was monitored using calipers, with volume calculated as volume = (length × width2)/2. For orthotopic models, minced tumor tissues from subcutaneous tumors were surgically implanted into the left hepatic lobe of C57BL/6 mice. To deplete CD8⁺ T cells in vivo, C57BL/6 mice received intraperitoneal injections of anti‐mouse CD8 monoclonal antibody (100 μg/100 μL PBS, Selleck, A2102, RRID: AB_3099521) or isotype control antibody (Selleck, A2116, RRID: AB_3662740) on Days 7, 11, 15, 19, and 23 post‐tumor implantations. Orthotopic tumor progression was tracked via bioluminescence imaging using an IVIS Spectrum system. Mice were intraperitoneally injected with 200 μL of 15 mg/mL d‐luciferin potassium salt (Beyotime, ST196), and fluorescence signals were acquired 15 min post‐injection to quantify tumor burden.

### Single‐cell transcriptomics

ScRNA‐seq data processing. In this study, we established orthotopic hepatocellular carcinoma models in C57BL/6 mice using Hepa1‐6‐WT and Hepa1‐6‐R132H cells, and outsourced single‐cell RNA sequencing (scRNA‐seq) of the samples to Hangzhou Cosmos Wisdom Biotechnology Co., Ltd. Single‐cell transcriptomic libraries were constructed using the MGI DNBelabC‐TaiM4RS high‐throughput single‐cell sequencing system, paired with the MGI DNBelab C Series High‐throughput Single‐cell RNA Library Preparation Set V3.0 (TaiM4) kit (MGI). Sequences were aligned to the human reference genome (GRCh38‐2020‐A) using CellRanger v7.2.0 to generate feature barcode matrices. Downstream sample analysis was performed per cell across all samples, culminating in merged feature barcode matrices for pooled samples. Subsequently, we employed Scanpy (version 1.8.2; https://scanpy.readthedocs.io/en/stable/) for further cell filtering, data normalization, cell subgroup classification, differential gene expression analysis, and marker gene calculation. High‐quality cells were filtered based on the following criteria: mitochondrial gene proportion (%) ≤10%, number of unique molecular identifiers detected per cell ≥500, and number of genes detected per cell ranging from 500 to 6000. The filtered expression matrix was processed with Scanpy for dimensionality reduction and clustering, with batch effects corrected using the default‐parameter BBKNN (Batch Balanced K Nearest Neighbors) algorithm. Data were further reduced via principal component analysis and visualized using UMAP. After defining major cell clusters, we extracted cells of the same type for re‐clustering with UMAP, graph‐based clustering, and marker gene analysis to refine cell type identification.

Gene functional enrichment analyses. Gene functional enrichment analyses, including GO analysis and GSEA, were conducted on differentially expressed genes using the clusterProfiler (v4.0.5) package. Terms with an adjusted *p*‐value < 0.05 (Benjamini–Hochberg correction) were selected and visualized via the ggplot2 (v3.3.6) package.

Cell‐cell communication analysis. To compare the cell–cell communication among different groups, CellChat (v1.4.0) was employed. For the scRNA‐seq dataset, the normalized gene expression matrices of WT, R132H, and R132H‐Flc‐L groups were input into the CellChat object. After merging the three groups into an integrated object using the merge CellChat function, differentially expressed pathways were identified for WT versus R132H and R132H‐Flc‐L versus R132H groups via the rankNet function. Ligand–receptor pairs were visualized as bubble plots using the netVisual_bubble function, with significant pairs defined by a corrected *p*‐value < 0.05.

### CD8^+^ T‐cell‐mediated tumor killing assay

C57BL/6 mice (6–8 weeks old, male) were sacrificed, and the spleen was isolated sterilely. The spleen was ground through a 70‐μm cell strainer in cold PBS to prepare a single‐cell suspension, which was centrifuged at 300 × *g* for 5 min. The pellet was resuspended in RBC lysis buffer (Beyotime, C3702) and incubated at room temperature for 5 min, then terminated with cold PBS. After centrifugation at 300 × *g* for 5 min, the pellet was washed twice with cold PBS to obtain splenocytes. CD8^+^ T cells were enriched using a mouse CD8^+^ T cell isolation kit (Yeasen, 37661ES50). The enriched CD8^+^ T cells were resuspended in complete RPMI‐1640 medium supplemented with recombinant mouse IL‐2 (Yeasen, 90142ES08) and anti‐mouse CD3/CD28 antibody‐conjugated magnetic beads (bead‐to‐cell ratio of 1:1, 11456D, Thermo Fisher). The cells were then cultured for 7 days to activate and expand them, with half of the medium being replaced every 2 days and supplemented with recombinant mouse IL‐2 to a final concentration of 500 U/mL. This process yielded activated and expanded CD8^+^ effector T cells. Hepa1‐6 cells were seeded into 12‐well plates at a density of 3 × 10⁵ cells per well and incubated overnight to allow for attachment. Prior to co‐culture, a co‐culture system was established using Transwell inserts. The lower chamber was seeded with adherent Hepa1‐6 cells, while activated CD8^+^ T cells were added to the upper chamber. The co‐culture was maintained for 48 h. Following co‐culture, the T cells in the upper chamber were gently aspirated, and the adherent Hepa1‐6 cells in the lower chamber were washed twice with PBS. Subsequently, the cell layer was covered with a 1% crystal violet solution and stained at room temperature for 10 min, followed by rinsing with tap water until no color was released. After air‐drying, the dye was dissolved in a 33% acetic acid solution. The solution was then transferred to a 96‐well plate, and the absorbance was measured at 570 nm using a microplate reader.

### Flow cytometry

Flow Cytometry Analysis of Tumor‐Infiltrating Immune Cells. Samples for flow cytometry were obtained from tumor‐bearing model mice and placed in OMICS‐Guard Sample Preservation Buffer (BD Biosciences, 570911). The tumors were minced in digestion solution containing collagenase (Solarbio, C8160), DNase type IV (Sigma Aldrich, D5025), hyaluronidase type V (Sigma‐Aldrich, H3884), and Hank's solution (Solarbio, H1025) to obtain a single‐cell suspension, which was percolated through 70 μm nylon strainers (Sangon, F613462). For extracellular staining, a mixed antibody panel (CD45 Antibody, 53‐0451‐82, Thermo Fisher, RRID: AB_2848416; CD3 Antibody, 47‐0031‐82, Thermo Fisher, RRID: AB_11149861; CD8 Antibody, 406‐0081‐82, Thermo Fisher, RRID: AB_2937150; IFN gamma Antibody, 12‐7311‐82, Thermo Fisher, RRID: AB_466193; PD‐1 Antibody, 17‐9981‐82, Thermo Fisher) was incorporated into the cell suspension at appropriate dilutions, reacted for half an hour at room temperature, and shielded from light. To stain the intracellular targets, a Fixation/Permeabilization Kit (Thermo Fisher, GAS001S100) was used to permeabilize the membrane. The corresponding intracellular fluorescent dyes were then added, and the cells were maintained for half an hour at 4°C in the dark. The samples were analyzed via a FACSVerse flow cytometer (Beckman Coulter), and the data were processed with FlowJo V10.8 (FlowJo LLC). The gating tactics are shown in Figure S11B. In vitro cytotoxicity assay. To assess the cytotoxic effect of CD8⁺ T cells, Hepa1‐6‐R132H cells were co‐cultured with or without CD8⁺ T cells in the presence or absence of 10 μM Flc for 48 h. After co‐culture, cells were stained with SYTOX Blue dead cell stain (Thermo Fisher, S34857) to label dead cells and analyzed by flow cytometry. To Detection of H‐2Kb surface expression, Hepa1‐6‐R132H cells were treated with increasing concentrations of Flc (0, 2.5, 5, 10 μM) for 48 h. Cells were then stained with an antibody against H‐2Kb‐APC (17‐5958‐82, Thermo Fisher, RRID: AB_1311280). Mean fluorescence intensity (MFI) was measured by flow cytometry and normalized to the corresponding IgG2a kappa Isotype Control (17‐4724‐81, Thermo Fisher, AB_470188).

### Quantification and statistical analysis

Data are presented as the mean ± standard error of the mean (SEM) for normally distributed variables or as the median with interquartile range for non‐normally distributed variables, as assessed by the Shapiro–Wilk or Kolmogorov–Smirnov test. A two‐sided *p* < 0.05 was considered statistically significant unless otherwise specified. The experimental unit for biological replicates was defined as data obtained from independent biological sources, and measurements from technical replicates were averaged to yield a single value per biological sample. For comparisons between two independent groups, an independent‐sample *t*‐test (for normal data) or the Mann–Whitney *U* test (for non‐normal data) was used. For comparisons within paired samples, the paired *t*‐test or the Wilcoxon signed‐rank test was applied for normal and non‐normal data, respectively. For comparisons among three or more groups, one‐way ANOVA (for parametric data) or the Kruskal–Wallis test (for non‐parametric data) was performed. For post hoc pairwise comparisons following a significant ANOVA, Tukey's honestly significant difference test was used, while Dunn's test with Bonferroni adjustment was applied following the Kruskal–Wallis test. For data with nested structures (e.g., multiple spots per sample on a protein chip), linear mixed‐effects models were employed to account for intra‐sample correlation. For the analysis of repeated‐measures data (e.g., tumor growth or proliferation over time), linear mixed‐effects models with subject‐specific random intercepts were fitted to account for the correlation of measurements within the same experimental unit over time. For controlling the false discovery rate (FDR) in high‐throughput analyses (including the initial screening of plant extracts, single‐cell RNA‐seq differential expression, and functional enrichment analyses), the Benjamini–Hochberg method was applied, with an FDR‐adjusted *p*‐value of less than 0.05 considered significant. For assessing correlations between two continuous variables, Pearson's correlation coefficient (for normal data) or Spearman's rank correlation coefficient (for non‐normal data) was used.

## AUTHOR CONTRIBUTIONS

**Zhao Cui**: Conceptualization; methodology; software; writing—review and editing; writing—original draft; funding acquisition; validation; resources. **Jiameng Li**: conceptualization; methodology; writing—original draft. **Caifeng Li**: Conceptualization; methodology; validation; software. **Mo Sun**: Software; validation; visualization. **Wei Liu**: Conceptualization; supervision; data curation. **Xuxia Cao**: Conceptualization; methodology; validation; investigation. **Shiwen Deng**: Conceptualization; methodology; investigation; data curation. **Junxian Cao**: Methodology; software; data curation. **Hongjun Yang**: Conceptualization; funding acquisition; project administration; supervision; investigation; writing—review and editing; resources. **Peng Chen**: Conceptualization; investigation; funding acquisition; writing—review and editing; project administration; supervision; resources. All authors have read the final manuscript and approved it for publication.

## CONFLICT OF INTEREST STATEMENT

The authors declare no conflicts of interest.

## ETHICS STATEMENT

The ethics application of animal studies (No. ERCCACMS21‐2504‐03) was approved by the Institutional Animal Care and Use Committee of the Experimental Research Center, China Academy of Chinese Medical Sciences.

## Supporting information


**Figure S1.** Positive drug‐target protein binding validation in a gel‐based click chemistry competition assay.
**Figure S2.** Optimization of Ccc‐Chip conditions for mIDH1 chip screening.
**Figure S3.** Determination of ΔT_m_ threshold for mIDH1 protein in DSF screening.
**Figure S4.** Effect of candidate extracts on cell proliferation.
**Figure S5.** HPLC of *P. methysticum* Forst and Flc.
**Figure S6.** Covalent binding of Flc to mIDH1 protein and cytotoxicity validation.
**Figure S7.** Effect of Flc on IDH1‐mutant xenograft tumor in nude mouse model.
**Figure S8.** *In vivo* safety evaluation of Flc.
**Figure S9.** Immunohistochemical analysis of immune cell landscape in nude mouse xenograft tumor.
**Figure S10.** Effect of Flc on H3K9 histone methylation and cGAS signaling pathway in orthotopic liver cancer in mice.
**Figure S11.** Co‐culture experiment validating Flc‐enhanced CD8⁺ T cell tumor‐killing activity and CD8⁺ T cell flow cytometry gating strategy.

**Table S1.** Comprehensive Comparison of Covalent Screening Strategies with Emphasis on Ccc‐Chip's High‐Throughput and Multi‐Target Capabilities.
**Table S2.** Effect of medicinal plant extracts on *T_m_* value of mIDH1 protein.
**Table S3.** Covalent virtual docking score.

## Data Availability

The data that support the findings of this study are openly available in Genome Sequence Archive (GSA) at https://ngdc.cncb.ac.cn/gsub/submit/bioproject/PRJCA053847/overview, reference number CRA036079. All the sequencing data have been deposited in GSA under submission number CRA036079, BioProject accession number PRJCA053847 (https://ngdc.cncb.ac.cn/gsub/submit/bioproject/PRJCA053847/overview). The data and scripts used are saved in GitHub https://github.com/CHUNYINCHO/IDH1imeta. Supplementary materials may be found in the online DOI or iMeta Science http://www.imeta.science/.
