# Supplementary material for: Construction of a clickable probe‐based protein chip platform for discovering covalent mIDH1 inhibitors from natural medicinal extracts
Source: Imeta. 2026 Feb 12;5(1):e70107. doi: 10.1002/imt2.70107 (PMC13726586; doi:10.1002/imt2.70107)
Supplement: Supplementary file 1 [file IMT2-5-e70107-s001.docx]

**Supporting information to**

**Construction of a Clickable Probe-Based Protein Chip Platform for Discovering Covalent mIDH1 Inhibitors from Natural Medicinal Extracts**

Zhao Cui^1,2,3#^ Jiameng Li^4,5#^, Caifeng Li^6#^, Mo Sun^7#^, Wei Liu^2^, Xuxia Cao^8^, Shiwen Deng^2^, Junxian Cao^2^, Hongjun Yang^2^*, Peng Chen^2^*

^1^National Clinical Research Center for TCM Cardiology, Xiyuan Hospital of China Academy of Chinese Medical Sciences, Beijing, China

^2^Experimental Research Center, China Academy of Chinese Medical Sciences, Beijing, China

^3^Laboratory of Cardiovascular Diseases, Xiyuan Hospital of China Academy of Chinese Medical Sciences, Beijing, China

^4^The First Affiliated Hospital of Guangzhou Medical University, Guangzhou, China

^5^CAS Key Laboratory for Biomedical Effects of Nanomaterials and Nanosafety, Chinese Academy of Sciences and National Center for Nanoscience and Technology of China, Beijing, China

^6^Jiangxi Province Key Laboratory of Traditional Chinese Medicine Pharmacology, Institute of Traditional Chinese Medicine Health Industry, China Academy of Chinese Medical Sciences, Nanchang, China

^7^School of Biological Sciences, Georgia Institute of Technology, Atlanta, USA.

^8^BinZhou Medical University, Yantai, China

^#^These authors contributed equally: Zhao Cui, Jiameng Li, Caifeng Li, and Mo Sun.

*Correspondences: sdzpchenpeng@qq.com (Peng Chen), hjyang@icmm.ac.cn (Hongjun Yang)

**Figure S1** **Positive drug-target protein binding validation in a gel-based click chemistry competition assay.** (A) In-gel fluorescence analysis of the competitive binding of Andro and IAA-yne to the recombinant P50 protein. IAA served as a positive control (*n* = 3). (B) In-gel fluorescence analysis of the competitive binding of Boru and IAA-yne to the recombinant AKT1 protein. IAA served as a positive control (*n* = 3). (C) In-gel fluorescence analysis of the competitive binding of AMG510 and IAA-yne to the recombinant KRAS^G12C^ protein. IAA served as a positive control (*n* = 3). Data are represented as mean ± SEM. **p* < 0.05; ***p <* 0.01; ****p* < 0.001.

**Figure S2** **Optimization of Ccc-Chip conditions for mIDH1 chip screening.** (A) Schematic diagram and signal curve for the signal-to-noise ratio (SNR) detection of the protein chip. The SNR is calculated by measuring the protein signal and background signal along a straight line (*n* = 2). (B) Schematic diagram for protein signal detection and the signal intensity at various protein concentrations (*n* = 2). (C) Correlation between protein signal and protein concentration under different concentrations of IAA-yne and click solution (*n* = 4). (D) Degree of competition between IAA and IAA-yne and the stability of the competitive signal under different protein concentration conditions *(n* = 4).

**Figure S3** **Determination of ΔT_m_ threshold for mIDH1 protein in DSF screening.** (A) Melting curves of mIDH1 protein after incubation with non-covalent binding positive molecules AG881, AG120, FT2102, and covalent binding positive molecules Scu, Cpd13. The combination of Scu and IDH1-C297S served as the negative control. (B) Changes in T_m_ values of mIDH1 protein following interaction with non-covalent binding positive molecules AG881, AG120, FT2102, and covalent binding positive molecules Scu, Cpd13. The combination of Scu and IDH1-C297S was used as the negative control (*n =* 3).

**Figure S4** **Effect of candidate extracts on cell proliferation.** The 24-hours proliferation curve of TS603 and HT1080 cell lines treated with candidate liquid-phase preparation components (*n* = 3).

**Figure S5** **HPLC of *P. methysticum* Forst and Flc.**

**Figure S6** **Covalent binding of Flc to mIDH1 protein and cytotoxicity validation.** (A) CETSA detected the influence of 10 μM Flc on the stability of IDH1-R132H in TS603 cell lysate. Β-actin was used as the negative control (*n* = 3). (B) Absorption spectra of Scu at different concentrations (*n* = 3). (C, D) BLI experiments to assess the affinity between Flc and IDH1-WT (C), or the IDH1-R132C (D) (*n* = 3). (E, F) The 24-hours proliferation curve of different cell lines treated with Flc or AG120 (*n* = 3).

**Figure S7** **Effect of Flc on IDH1-mutant xenograft tumor in nude mouse model.** (A) Schematic plan for the administration of AG120 (40 mg/kg/day) and Flc (60 and 120 mg/kg/day) in a TS603 cell subcutaneous mouse model. (B-E) Representative images (C), tumor weights (D), growth curves (E), and body weights (B) of a subcutaneous tumor model established with the TS603 cell and nude mice (*n* = 7). (F, G) Ki67 and 5-hmC IHC analysis of TS603 cell subcutaneous nude mice model (*n* = 6). (H, I) WB analysis of histone H3 methylation, cGAS, STING, and p-IRF3/IRF3 of TS603 cell subcutaneous nude mice model (*n* = 4). Data are represented as mean ± SEM. **p* < 0.05; ***p <* 0.01; ****p* < 0.001; *ns*, not significant.

**Figure S8** ***In vivo* safety evaluation of Flc.** Histological examination of the major organs (heart, liver, spleen, lungs and kidneys) of TS603 cell subcutaneous nude mice was performed using vehicle control and Flc (120 mg/kg/day, 3 weeks).

**Figure S9** **Immunohistochemical analysis of immune cell landscape in nude mouse xenograft tumor.** CD4, CD8, CD56, and F4/80 IHC analysis of the TS603 cell subcutaneous nude mice model.

 **Figure S10** **Effect of Flc on H3K9 histone methylation and CGAS signaling pathway in orthotopic liver cancer in mice.** (A) Detecting the expression of IDH1-R132H after stable overexpression of IDH1-R132H in hepa1-6 cells by western blotting. (B) Cell markers of total clusters used for annotating cell types in scRNA-seq analysis. (C) WB analysis of histone H3 methylation of TS603 cell subcutaneous nude mice model (*n* = 4). (D) Ki67, H3K9me3, cGAS, and CD8 IHC analysis of the orthotopic Hepa1-6-R132H-derived tumor model (*n* = 6). Data are represented as mean ± SEM. **p* < 0.05; ***p <* 0.01; ****p* < 0.001.

 **Figure S11** **Co-culture experiment validating Flc-enhanced CD8⁺ T cell tumor-killing activity and CD8⁺ T cell flow cytometry gating strategy.**  (A) Cell markers of subcluster of T/NK cells used for annotating cell types in scRNA-seq analysis. (B) Flow cytometry gating strategy used in this study that was used to sort total CD8^+^ T cells, IFN-γ^+^ CD8^+^ T cells, and PD1^+^ CD8^+^ T cells. (C, D) Schematic diagrams of the CD8^+^ T-cell mediated tumor killing assay (C), which was conducted by coculturing activated CD8^+^ T cells and hepa1-6 cells. Before cocultivation, activated CD8^+^ T cells were pretreated with supernatants from WT and R132H hepa1-6 cells treated with Flc (D) (*n* = 3). Data are represented as mean ± SEM. **p* < 0.05; ***p <* 0.01; ****p* < 0.001; *ns*, not significant.
